# Genome-wide comparison reveals divergence of cassava and rubber aquaporin family genes after the recent whole-genome duplication

**DOI:** 10.1186/s12864-019-5780-4

**Published:** 2019-05-15

**Authors:** Zhi Zou, Jianghua Yang

**Affiliations:** 1 Key Laboratory of Biology and Genetic Resources of Tropical Crops, Ministry of Agriculture and Rural Affairs, Institute of Tropical Biosciences and Biotechnology, Chinese Academy of Tropical Agricultural Sciences, Haikou, 571101 Hainan People’s Republic of China; 2 Rubber Research Institute, Chinese Academy of Tropical Agricultural Sciences, Haikou, 571101 Hainan People’s Republic of China

**Keywords:** Aquaporin, *AQP* gene family, Gene duplication, Expansion, Evolution, Orthologous group, Phylogenetic analysis, Whole-genome duplication

## Abstract

**Background:**

Aquaporins (AQPs) are a class of integral membrane proteins that facilitate the passive transport of water and other small solutes across biological membranes. Despite their importance, little information is available in cassava (*Manihot esculenta*), a perennial shrub of the Euphorbiaceae family that serves the sixth major staple crop in the world.

**Results:**

This study presents a genome-wide analysis of the *AQP* gene family in cassava. The family of 42 members in this species could be divided into five subfamilies based on phylogenetic analysis, i.e., 14 plasma membrane intrinsic proteins (PIPs), 13 tonoplast intrinsic proteins (TIPs), nine NOD26-like intrinsic proteins (NIPs), four X intrinsic proteins (XIPs), and two small basic intrinsic proteins (SIPs). Best-reciprocal-hit-based sequence comparison and synteny analysis revealed 34 orthologous groups (OGs) present in the Euphorbiaceae ancestor, and nearly one-to-one or two-to-one orthologous relationships were observed between cassava with rubber/physic nut, reflecting the occurrence of one so-called ρ recent whole-genome duplication (WGD) in the last common ancestor of cassava and rubber. In contrast to a predominant role of the ρ WGD on family expansion in rubber, cassava *AQP* duplicates were derived from the WGD as well as local duplication. Species-specific gene loss was also observed in cassava, which includes the entire NIP4 group and/or six OGs. Comparison of conserved motifs and gene expression profiles revealed divergence of paralogs in cassava as observed in rubber.

**Conclusions:**

Our findings will not only improve our knowledge on family evolution in Euphorbiaceae, but also provide valuable information for further functional analysis of *AQP* genes in cassava and rubber.

**Electronic supplementary material:**

The online version of this article (10.1186/s12864-019-5780-4) contains supplementary material, which is available to authorized users.

## Background

Cassava (*Manihot esculenta* Crantz, *2n* = 36) is a perennial shrub that belongs to Euphorbiaceae, one of the largest plant families also including rubber (*Hevea brasiliensis* Muell. Arg., *2n* = 36), castor (*Ricinus communis* L., *2n* = 20), and physic nut (*Jatropha curcas* L., *2n* = 22) [1–6]. Cassava was domesticated from its wild progenitor, *M. esculenta* ssp. *flabellifolia*, along the southern border of the Amazon basin [7]. Now, cassava is widely cultivated in tropical regions and represents the sixth major staple crop in the world [8, 9]. Besides servicing as human foods and livestock feeds, the starchy-enriched storage root of cassava is ideal for bio-ethanol production [10]. The cassava genome was estimated to be 772 Mb and three assemblies have been available for three lines: W14 (*M. esculenta* ssp. *flabellifolia*), a wild subspecies with low storage root yield and low root starch content; KU50 (also known as MTAI16), a widely cultivated variety with high storage root yield, high starch content, and vigorous plant growth with wide adaptability to unfavourable conditions; and, AM560–2, a partial inbred line of MCOL1505 [8, 9]. The most complete one (i.e. AM560–2) spans about 582 Mb and 89.0% of this assembly could be anchored to 18 chromosomes (Chrs) based on 22,403 genetic markers available [9]. In addition to the ancient so-called γ whole-genome triplication shared by all core eudicots, comparative genomics analysis showed that the ancestor of cassava experienced one recent whole-genome duplication (WGD, named ρ in this study) after its divergence with *Ricinus* and *Jatropha* [5, 6, 11, 12]. This WGD was estimated to occur within a window of 39–47 million years ago (Mya), which is shared by *Hevea* [2, 9, 11, 12]. Despite sharing the same recent WGD, the morphology of rubber, which is characterized as a perennial big tree, is obviously distinct from cassava [5, 6, 11–13]. Moreover, rubber was shown to harbor a considerably bigger genome size, i.e. approximate 2.15 or 1.5 Gb inferred from Feulgen microdensitometry and sequencing-based K-mer analysis, respectively [14, 15]. According to the most complete assembly (i.e. Reyan7–33-97) that spans about 1.37 Gb, the number of protein-coding genes in rubber was shown to be 43,792 which is relatively more than 33,033 in cassava [9, 15], suggesting different fates of duplicated genes after the ρ WGD. Thereby, it is of particular interest to study the evolutionary fate of duplicated genes in these two special species.

Aquaporins (AQPs), a special class of integral membrane proteins in the ancient major intrinsic protein (MIP) superfamily, are distributed in all types of organisms, including microbes, animals, and plants [1, 16–18]. AQPs are characterized by six transmembrane helices (i.e. TM1–TM6) connected by five loops (i.e. LA–LE), two short helices (i.e. HB and HE), two NPA (Asn-Pro-Ala) motifs, and the ar/R (aromatic/arginine) selectivity filter (i.e. H2, H5, LE1, and LE2) [19, 20]. In addition to water, some AQP family members also transport other small solutes, e.g. glycerol, urea, boric acid, silicic acid, arsenic, ammonia (NH_3_), carbon dioxide (CO_2_), oxygen (O_2_), and hydrogen peroxide (H_2_O_2_), where glycerol facilitators were called aquaglyceroporins (GLPs) [16, 19, 21]. Compared with few members present in microbes and animals, the AQP family was shown to have particularly expanded in high plants, which can be divided into five main subfamilies based on sequence similarity: plasma membrane intrinsic proteins (PIPs), tonoplast intrinsic proteins (TIPs), NOD26-like intrinsic proteins (NIPs), small basic intrinsic proteins (SIPs), and X intrinsic proteins (XIPs) [1, 17, 18, 22–24]. The former four subfamilies are widely distributed, whereas XIPs are absent from monocots and the Brassicaceae family in dicots [17, 25, 26]. The fast expansion of this gene family is usually associated with several WGD events, e.g. the γ event for core eudicots and the τ event for monocots [27, 28]. Moreover, it is well established that arabidopsis (*Arabidopsis thaliana*) experienced two additional doubling events, known as β and α, respectively [29], whereas poplar (*Populus trichocarpa*) experienced one Salicaceae-specific recent WGD [30]. As a result, a high number of *AQP* gene pairs (i.e. paralogs) were identified in these two species [1, 23, 24]. For example, 35 arabidopsis *AQP* genes were shown to result from 18 parents, including eight or two *AQP* genes from α and β WGDs, respectively [31]. Genome-wide comparison of rubber, castor and, physic nut *AQP* genes also revealed a high number of duplicates present in rubber, and the pattern is highly similar to poplar [1, 18]. Nevertheless, the origin of rubber *AQP* duplicates was not well resolved due to the lack of a high-density genetic map [1, 24]. The recently available chromosome-scale structure of the cassava genome allows us to address this issue. In this paper, we report a genome-wide identification and manual curation of *AQP* family genes in cassava by using available genome and transcriptome datasets. Moreover, we would also like to present a comprehensive comparison of cassava and rubber *AQP* genes based on analysis of gene structures, sequence characteristics, orthologous relationships, and expression profiles.

## Methods

### Datasets and sequence retrieval

*AQP* genes reported in rubber, castor, physic nut, and poplar were obtained according to related literatures, and accession numbers can be found in Additional file 1. The cassava genome sequences were downloaded from Phytozome v12 (https://phytozome.jgi.doe.gov/pz/portal.html), whereas other data such as nucleotides, Sanger expressed sequence tags (ESTs) and RNA sequencing (RNA-seq) reads were all accessed from NCBI (https://www.ncbi.nlm.nih.gov/).

### Identification and manual curation of *AQP* family genes in cassava

MeAQP proteins available in GenBank were used as queries to search for homologs from the cassava genome. The E-value in the tBLASTn search [32] was set to 1e-5, and positive genomic sequences were predicted as described before [1, 24]. Predicted gene models were further validated with ESTs and RNA-seq reads when available. Homology search for nucleotides or ESTs was performed using BLASTn [32]. RNA-seq data were also adopted for the expression annotation as described before [24], where read alignment was performed using Bowtie 2 [33].

### Synteny analysis and gene expansion patterns

The all-to-all BLASTP was used to identify homolog pairs as described before [5, 6]. Syntenic blocks and gene collinearity were inferred using MCScanX [34]. WGD duplicates were defined when duplicated genes are located in syntenic blocks of duplicated chromosomes, while tandem duplications were considered when two duplicated genes were consecutive in a genome. For duplicate pairs, Ka (nonsynonymous substitution rate) and Ks (synonymous substitution rate) were calculated by codeml in the PAML package [35].

### Sequence alignment, phylogenetic analysis, and classification

Multiple sequence alignment of full-length AQP proteins was performed using MUSCLE [36]. Unrooted trees were constructed using MEGA 6.0 [37] with the maximum likelihood method, where the bootstrap was set to 1000 replicates. Classification of AQPs into subfamilies and groups was done as previously described [23]. Orthologous groups (OGs) across different species were inferred from BRH (best reciprocal hit)-based sequence comparison as described before [2, 11, 12]. As for cassava and rubber, information from results of above synteny analysis was also considered.

### Structural features of MeAQPs

Protein features such as theoretical molecular weight (MW), isoelectric point (*p*I), and grand average of hydropathicity (GRAVY) were calculated using ProtParam (https://web.expasy.org/protparam/). Functional prediction was performed based on analysis of dual NPA motifs, ar/R filter, and five Froger’s positions (five conserved residues named P1–5 for discriminating GLPs from water-conducting AQPs) from alignments with the structure resolved spinach (*Spinacia oleracea*) PIP2;1 and AtTIP2;1 as well as functionally characterized AQPs [20, 38, 39]. Additionally, conserved motifs in Me/HbAQP proteins were analyzed using MEME [40], and optimized parameters were as follows: any number of repetitions; maximum number of motifs, 25; and, the optimum width of each motif, between 6 and 50 residues. The MAST program [41] was also used to search detected motifs in protein databases.

### Gene expression analysis

Global gene expression profiles of *MeAQP* genes were investigated over various tissues as described before (GEO accession number GSE82279) [42], i.e. shoot apical meristem (SAM), lateral bud, leaf blade, leaf midvein, petiole, stem, fibrous root, storage root, root apical meristem (RAM), friable embryogenic callus (FEC), and somatic organized embryogenic structure (OES): 101 paired-end reads were generated using Illumina HiSeq 2500, and three biological replicates were performed for most tissues except for storage root with two replicates. Raw reads were first filtered by removing adaptor sequences, adaptor-only reads, and low quality reads containing more than 50% bases with Q-value ≤5. Obtained clean reads were mapped to identified *MeAQPs* and other protein-coding genes using Bowtie 2 [33], and the FPKM (fragments per kilobase of exon per million fragments mapped) method [43] was used for determination of transcript levels. Unless specific statements, tools used in this study were performed with default parameters.

## Results

### Characterization of 43AQP-encoding loci in cassava

The search of the cassava genome resulted in 42 AQP-coding genes (Table 1), corresponding to 43 loci reported by the genome annotation [9]. Among them, *MeSIP2;1* (see Additional file 2), spanning 17,010 bp that was supported by three ESTs and thousands of RNA-seq reads, was annotated as two loci, i.e. Manes.09G074100 and Manes.09G074000. Moreover, based on expert revision of gene structures via aligning ESTs and reads to AQP-coding genome sequences, the gene models of three other loci (i.e. Manes.16G044000, Manes.11G089200, and Manes.11G089100) were also optimized (see Additional files 3, 4 and 5).

**Table 1:** Cassava *AQP* family genes identified in this study

Gene name	Locus ID	Chr location	Nucleotide length (bp, from start to stop codons)		Intron no.	EST no.	Comment
			CDS	Gene			
*MePIP1;1*	Manes.01G059600	Chr1:17535987–17,539,206	864	2626	3	15	
*MePIP1;2*	Manes.02G020100	Chr2:1624224–1,626,227	864	1654	3	16	
*MePIP1;3*	Manes.07G126600	Chr7:25215682–25,217,264	864	1157	3	20	
*MePIP1;4*	Manes.10G016600	Chr10:1312629–1,314,244	864	1166	3	23	
*MePIP2;1*	Manes.08G006800	Chr8:512954–514,468	867	1127	3	21	
*MePIP2;2*	Manes.09G068800	Chr9:9307274–9,308,662	867	1157	3	4	
*MePIP2;3*	Manes.07G100500	Chr7:22842955–22,845,156	858	1852	3	3	
*MePIP2;4*	Manes.10G046200	Chr10:4375805–4,379,849	861	3655	3	5	
*MePIP2;5*	Manes.04G021200	Chr4:2304659–2,306,481	861	1396	3	2	
*MePIP2;6*	Manes.11G145300	Chr11:25677637–25,679,377	861	1300	3	10	
*MePIP2;7*	Manes.04G076500	Chr4:21375880–21,377,507	843	1265	3	21	
*MePIP2;8*	Manes.11G096600	Chr11:15805567–15,807,131	843	1236	3	2	
*MePIP2;9*	Manes.02G109200	Chr2:8173111–8,174,880	852	1330	3	17	
*MePIP2;10*	Manes.05G144100	Chr5:20526797–20,528,273	846	1122	3	3	
*MeTIP1;1*	Manes.08G012800	Chr8:924654–925,957	759	855	1	47	
*MeTIP1;2*	Manes.09G062300	Chr9:8402765–8,403,903	759	853	1	80	
*MeTIP1;3*	Manes.07G111500	Chr7:23996724–23,997,990	759	957	2	0	
*MeTIP1;4*	Manes.10G035000	Chr10:2983924–2,985,053	759	848	1	2	
*MeTIP1;5*	Manes.04G030400	Chr4:3360155–3,361,296	759	962	2	0	
*MeTIP1;6*	Manes.11G134600	Chr11:24689956–24,691,006	759	977	2	1	
*MeTIP2;1*	Manes.11G036500	Chr11:3094579–3,095,833	747	954	2	17	
*MeTIP2;2*	Manes.01G081600	Chr1:20741570–20,742,776	753	928	2	1	
*MeTIP2;3*	Manes.02G040800	Chr2:3161794–3,163,035	753	969	2	7	
*MeTIP3;1*	Manes.01G160000	Chr1:26663550–26,664,500	777	951	2	0	
*MeTIP3;2*	Manes.02G118300	Chr2:8751200–8,752,174	777	975	2	0	
*MeTIP4;1*	Manes.03G062300	Chr3:6536427–6,538,114	744	1458	2	6	
*MeTIP5;1*	Manes.14G036400	Chr14:2919392–2,920,528	759	1137	2	0	
*MeNIP1;1*	Manes.16G044000	Chr16:6177753–6,179,082	840	1600	4	0	Misannotated
*MeNIP1;2*	Manes.17G061100	Chr17:19971252–19,973,361	858	1712	4	1	
*MeNIP2;1*	Manes.01G091200	Chr1:21577225–21,580,168	867	2458	4	2	
*MeNIP3;1*	Manes.02G152300	Chr2:11346718–11,348,621	852	1213	4	0	
*MeNIP3;2*	Manes.12G133900	Chr12:28849870–28,851,480	822	1102	4	0	
*MeNIP3;3*	Manes.13G093600	Chr13:19561358–19,562,924	825	1049	4	0	
*MeNIP5;1*	Manes.01G001400	Chr1:259425–262,398	897	2448	3	4	
*MeNIP6;1*	Manes.04G104100	Chr4:24004959–24,008,297	921	2638	4	0	
*MeNIP7;1*	Manes.03G183400	Chr3:27015982–27,017,206	900	1225	4	0	
*MeXIP1;1*	Manes.04G078900	Chr4:21735846–21,737,169	885	1234	1	0	
*MeXIP2;1*	Manes.11G089300	Chr11:12980304–12,982,216	915	1615	2	2	
*MeXIP3;1*	Manes.11G089200	Chr11:12950526–12,952,826	915	2301	2	0	Misannotated
*MeXIP3;2*	Manes.11G089100	Chr11:12935690–12,936,439	924	4465	2	0	Misannotated
*MeSIP1;1*	Manes.09G144400	Chr9:26227923–26,233,566	720	5135	2	5	
*MeSIP2;1*	Manes.09G074000	Chr9:10639757–10,640,714	714	17,010	2	3	Misannotated

(*bp* base pair, *CDS* coding sequence, *Chr* chromosome, *EST* expressed sequence tag)

Except for *MePIP2;7* (GenBank accession number EU599222), homology search showed that no full-length cDNA sequences of other 41 family genes have been reported in any public database (as of Dec 2017). Nevertheless, 28 members had EST hits in GenBank and *MeTIP1;2* was found to harbor the most of 80 hits (Table 1). Moreover, the expression of other family members was supported by available RNA-seq reads derived from various transcriptomes of somatic embryo, embryogenic callus, embryogenic structure, leaf blade, leaf midvein, petiole, stem, SAM, lateral bud, fibrous root, storage root, and RAM.

**Fig. 1:**
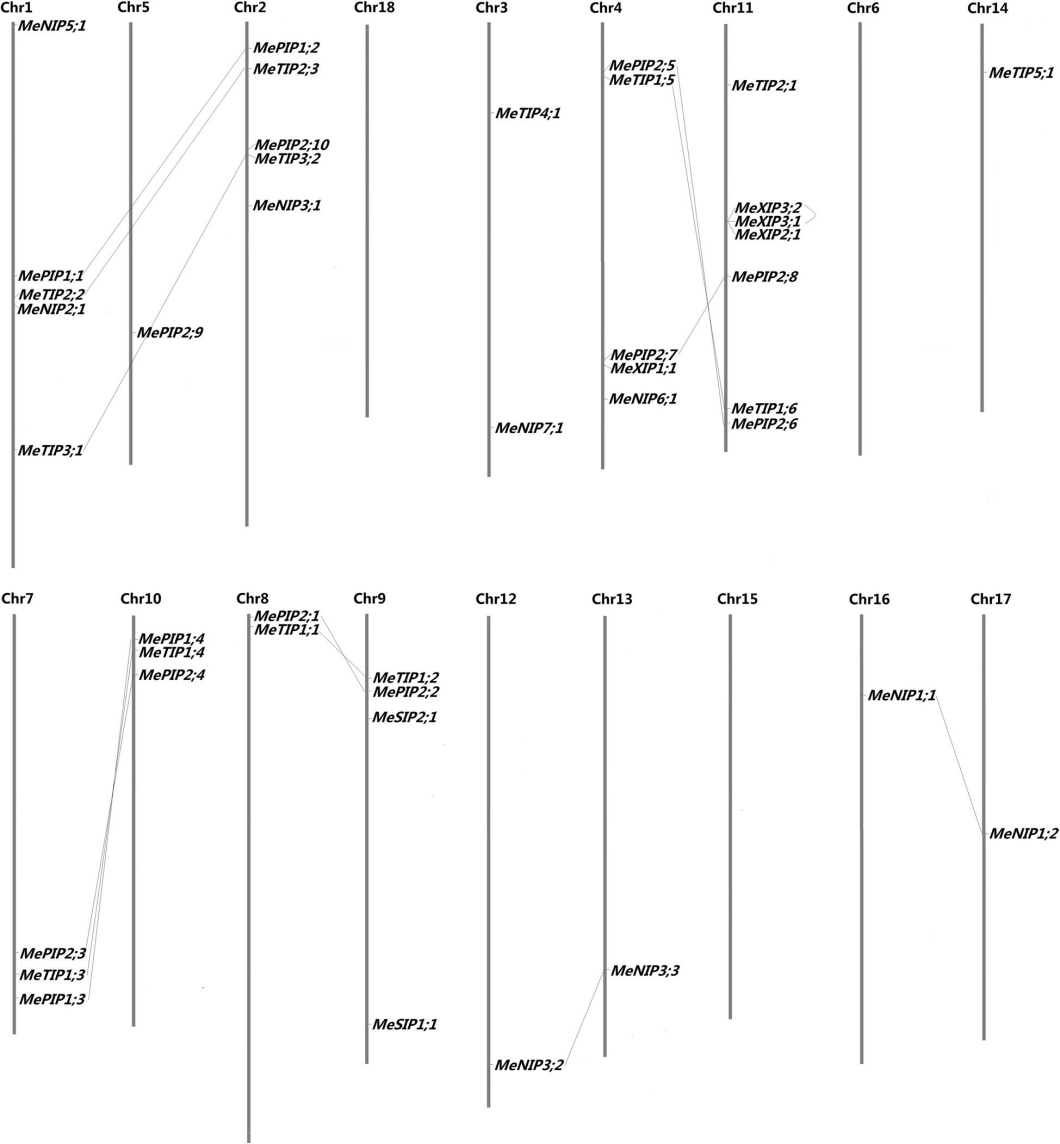
Chromosomal locations and duplication events of 42 *MeAQP* genes. The chromosome serial number is indicated at the top of each chromosome. *MeXIP3;1* and *MeXIP3;2* are clustered as tandem duplication, and the lines connect the corresponding 13 duplication pairs in syntenic blocks

These *MeAQPs* were found to locate on 15 out of the 18 chromosomes, only excluding Chromosomes 6, 15, and 18 (Fig. 1). The gene distribution looks uneven: six chromosomes (counting 40.0%) harbor a single *AQP* gene, whereas Chromosome 11 contains the most of seven genes. As shown in Table 2, the CDS (coding sequences) of 14 gene pairs exhibit a relatively high identity at the nucleotide level, varying from 81.7 to 93.5%. *MeXIP3;1*/*− 3;2* can be defined as tandem duplication for their adjacent organization on the same chromosome. By contrast, other gene pairs are located in syntenic blocks of duplicated chromosomes and thus were considered to result from the ρ WGD. The Ka/Ks ratios of these duplicates are all below one (from 0.0258 to 0.3751) (Table 2), suggesting that their divergence was driven by purifying selection.

**Table 2:** Cassava *AQP* duplicates identified in this study

Duplicated gene pair	Identity (%)	Ks	Ka/Ks
*MePIP1;1*/*MePIP1;2*	91.0	0.3169	0.0807
*MePIP1;3*/*MePIP1;4*	93.5	0.2442	0.0546
*MePIP2;1*/*MePIP2;2*	91.0	0.3260	0.0876
*MePIP2;3*/*MePIP2;4*	88.2	0.4224	0.1089
*MePIP2;5*/*MePIP2;6*	89.3	0.5591	0.0258
*MePIP2;7*/*MePIP2;8*	91.9	0.3106	0.0703
*MeTIP1;1*/*MeTIP1;2*	89.7	0.4090	0.0699
*MeTIP1;3*/*MeTIP1;4*	90.3	0.2952	0.1555
*MeTIP1;5*/*MeTIP1;6*	86.7	0.5756	0.0646
*MeTIP2;2*/*MeTIP2;3*	88.6	0.4055	0.0898
*MeTIP3;1*/*MeTIP3;2*	90.5	0.3015	0.1214
*MeNIP1;1*/*MeNIP1;2*	83.3	0.3958	0.2888
*MeNIP3;2*/*MeNIP3;3*	81.7	0.4731	0.2702
*MeXIP3;1*/*MeXIP3;2*	89.8	0.1877	0.3751

Ks and Ka were calculated using PAML. (*Ka* nonsynonymous substitution rate, *Ks* synonymous substitution rate)

### Phylogenetic analysis and classification

**Fig. 2:**
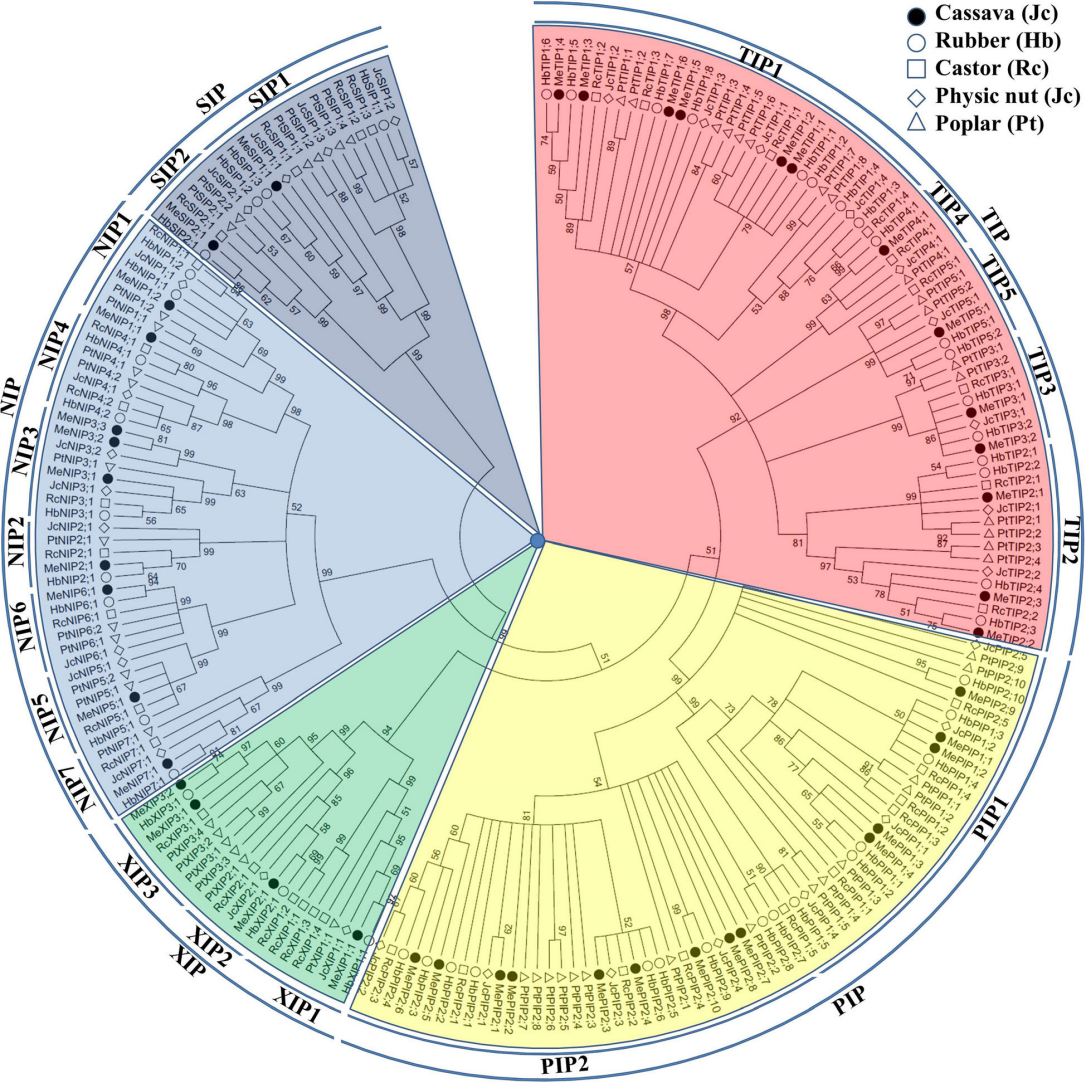
Phylogenetic analysis of cassava, rubber, castor, physic nut, and poplar AQPs. Sequence alignment was performed using MUSCLE and the unrooted phylogenetic tree was constructed using bootstrap maximum likelihood tree (1000 replicates) method of MEGA 6.0. The distance scale denotes the number of amino acid substitutions per site. The name of each subfamily/group is indicated next to the corresponding cluster

To analyze the evolutionary relationships and infer putative functions, an unrooted phylogenetic tree was constructed from all MeAQPs together with 48 HbAQPs, 37 RcAQPs, 31 JcAQPs, and 55 PtAQPs. Poplar, a representative plant of the Salicaceae family also belonging to the order Malpighiales as Euphorbiaceae, was used as an out-group of Euphorbiaceous plants. According to the tree, 42 MeAQPs were grouped into five subfamilies, i.e. PIP (14), TIP (13), NIP (9), SIP (2), and XIP (4). The PIP subfamily can be further divided into two phylogenetic groups (i.e. four MePIP1s and ten MePIP2s), the TIP subfamily into five groups (i.e. six MeTIP1s, three MeTIP2s, two MeTIP3s, one MeTIP4, and one MeTIP5), the NIP subfamily into six groups (two MeNIP1s, one MeNIP2, three MeNIP3s, one MeNIP5, one MeNIP6, and one MeNIP7), the SIP subfamily into two groups (one MeSIP1 and one MeSIP2), and the XIP subfamily into three groups (one MeXIP1, one MeXIP2, and two MeXIP3s) (Fig. 2). Interestingly, the widely distributed NIP4 group was not found in cassava, though genome sequences of W14 and KU50 and various transcriptome data were also mined.

The BRH-based sequence comparison as well as synteny analysis were also adopted to identify orthologous groups across cassava, rubber, castor, physic nut, and poplar. As shown in Table 3, a total of 34 OGs were identified and each phylogenetic group was shown to contain one to six OGs. It is worth noting that, species-specific gene expansion or loss was obviously observed, where only 28 OGs have retained in cassava (Table 3).

**Table 3:** 34 identified OGs based on comparison of five examined species

Group	OG	Cassava	Rubber	Castor	Physic nut	Poplar
PIP1	1-1a	*MePIP1;1*	*HbPIP1;4*	*RcPIP1;4*	*JcPIP1;2*	*PtPIP1;3*
		***MePIP1;2***	***HbPIP1;3***			
PIP1	1-1b	*MePIP1;3*	*HbPIP1;2*	*RcPIP1;2* *RcPIP1;3*	*JcPIP1;1*	*PtPIP1;1* *PtPIP1;2*
		***MePIP1;4***	***HbPIP1;1***			
PIP1	1-1c	*–*	*–*	*RcPIP1;1*	*–*	*PtPIP1;4* *PtPIP1;5*
PIP1	1-1d	*–*	*HbPIP1;5*	*RcPIP1;5*	*JcPIP1;4*	*–*
PIP2	1-2a	*MePIP2;1*	*HbPIP2;1*	*RcPIP2;1*	*JcPIP2;1*	*PtPIP2;5* *PtPIP2;6* *PtPIP2;7*
		***MePIP2;2***	***HbPIP2;2***			
PIP2	1-2b	*MePIP2;3*	*HbPIP2;5*	*RcPIP2;2*	*JcPIP2;2*	*PtPIP2;3* *PtPIP2;4*
		***MePIP2;4***	***HbPIP2;6***			
PIP2	1-2c	*MePIP2;5*	*HbPIP2;4*	*RcPIP2;3*	*JcPIP2;3*	*PtPIP2;8*
		***MePIP2;6***	***HbPIP2;3***			
PIP2	1-2d	*MePIP2;7*	*HbPIP2;7*	*RcPIP2;4*	*JcPIP2;4*	*PtPIP2;1* *PtPIP2;2*
		***MePIP2;8***	***HbPIP2;8***			
PIP2	1-2e	*MePIP2;9*	*HbPIP2;9*	*–*	*–*	*–*
PIP2	1-2f	*MePIP2;10*	*HbPIP2;10*	*RcPIP2;5*	*JcPIP2;5*	*PtPIP2;9* *PtPIP2;10*
TIP1	2-1a	*MeTIP1;1*	*HbTIP1;2*	*RcTIP1;1*	*JcTIP1;1*	*PtTIP1;5* *PtTIP1;6*
		***MeTIP1;2***	***HbTIP1;1***			
TIP1	2-1b	*–*	*HbTIP1;3* *HbTIP1;4*	*RcTIP1;4*	*JcTIP1;4*	*PtTIP1;7* *PtTIP1;8*
TIP1	2-1c	*MeTIP1;3*	*HbTIP1;6*	*RcTIP1;2*	*JcTIP1;2*	*PtTIP1;4* *PtTIP1;3*
		***MeTIP1;4***	***HbTIP1;5***			
TIP1	2-1d	*MeTIP1;5*	*HbTIP1;8*	*RcTIP1;3*	*JcTIP1;3*	*PtTIP1;1* *PtTIP1;2*
		***MeTIP1;6***	***HbTIP1;7***			
TIP2	2-2a	*MeTIP2;1*	*HbTIP2;1* *HbTIP2;2*	*RcTIP2;1*	*JcTIP2;1*	*PtTIP2;1* *PtTIP2;2*
TIP2	2-2b	*MeTIP2;2*	*HbTIP2;3*	*RcTIP2;2*	*JcTIP2;2*	*PtTIP2;3* *PtTIP2;4*
		***MeTIP2;3***	***HbTIP2;4***			
TIP3	2–3	*MeTIP3;1*	*HbTIP3;1*	*RcTIP3;1*	*JcTIP3;1*	*PtTIP3;2* *PtTIP3;1*
		***MeTIP3;2***	***HbTIP3;2***			
TIP4	2–4	*MeTIP4;1*	*HbTIP4;1*	*RcTIP4;1*	*JcTIP4;1*	*PtTIP4;1*
TIP5	2–5	*MeTIP5;1*	*HbTIP5;1* *HbTIP5;2*	*RcTIP5;1*	*JcTIP5;1*	*PtTIP5;1* *PtTIP5;2*
NIP1	3–1	*MeNIP1;1*	*HbNIP1;1*	*RcNIP1;1*	*JcNIP1;1*	*PtNIP1;1* *PtNIP1;2*
		***MeNIP1;2***	***HbNIP1;2***			
NIP2	3–2	*MeNIP2;1*	*HbNIP2;1*	*RcNIP2;1*	*JcNIP2;1*	*PtNIP2;1*
NIP3	3-3a	*MeNIP3;1*	*HbNIP3;1*	*RcNIP3;1*	*JcNIP3;1*	*PtNIP3;1*
NIP3	3-3b	*MeNIP3;2* *MeNIP3;3*	*–*	*–*	*JcNIP3;2*	*–*
NIP4	3-4a	*–*	*HbNIP4;1*	*RcNIP4;1*	*–*	*PtNIP4;1*
NIP4	3-4b	*–*	*HbNIP4;2*	*RcNIP4;2*	*JcNIP4;1*	*PtNIP4;2*
NIP5	3–5	*MeNIP5;1*	*HbNIP5;1*	*RcNIP5;1*	*JcNIP5;1*	*PtNIP5;1* *PtNIP5;2*
NIP6	3–6	*MeNIP6;1*	*HbNIP6;1*	*RcNIP6;1*	*JcNIP6;1*	*PtNIP6;1* *PtNIP6;2*
NIP7	3–7	*MeNIP7;1*	*HbNIP7;1*	*RcNIP7;1*	*JcNIP7;1*	*PtNIP7;1*
XIP1	4–1	*MeXIP1;1*	*HbXIP1;1*	*RcXIP1;1* *RcXIP1;2* *RcXIP1;3* *RcXIP1;4*	*JcXIP1;1*	*PtXIP1;1*
XIP2	4–2	*MeXIP2;1*	*HbXIP2;1*	*RcXIP2;1*	*JcXIP2;1*	*PtXIP2;1*
XIP3	4–3	*MeXIP3;1* *MeXIP3;2*	*HbXIP3;1*	*RcXIP3;1*	*–*	*PtXIP3;1* *PtXIP3;2* *PtXIP3;3*
SIP1	5-1a	*MeSIP1;1*	*HbSIP1;2* *HbSIP1;3*	*RcSIP1;1*	*JcSIP1;1*	*PtSIP1;1* *PtSIP1;2*
SIP1	5-1b	*–*	*HbSIP1;1*	*RcSIP1;2* *RcSIP1;3*	*JcSIP1;2* *JcSIP1;3*	*PtSIP1;3* *PtSIP1;4*
SIP2	5–2	*MeSIP2;1*	*HbSIP2;1*	*RcSIP2;1*	*JcSIP2;1*	*PtSIP2;1* *PtSIP2;2*

OGs that are limited to rubber and cassava were shown in black. (*OG* orthologous group)

### Analysis of exon-intron structure

The exon-intron structures of *MeAQP* genes were analyzed based on revised gene models. Compared with the ORF (open reading frame), the gene length (from start to stop codons) is considerably more variable, i.e. 848–17,010 bp vs 714–924 bp. The intron number of *MeAQP* genes varies from one to four, and the majority of them (accounting for 76.2%) contain two or three introns. The average intron length is about 423 bp, with the minimum of 71 bp for the second intron of *MeNIP3;2* and the maximum of 16,179 bp for the first intron of *MeSIP2;1*. The exon-intron structure is usually highly conserved in the same subfamily but distinct between different subfamilies: the PIP subfamily features three introns; the TIP subfamily features two introns except for three members (i.e. *MeTIP1;1*, *MeTIP1;2*, and *MeTIP1;4*) that harbor a single one; the NIP subfamily features four introns except for *MeNIP5;1* that harbors three introns; the SIP subfamily features two introns; and, the XIP subfamily features one (XIP1 group) or two (XIP2 and XIP3 groups) (Table 1).

### Structural features of MeAQPs

Sequence analysis showed that 42 MeAQPs consist of 237–307 amino acids (AA), with a theoretical molecular weight of 25.14–32.64 kDa and a *p*I value of 4.79–9.67 (Table 4). The GRAVY value was all shown to be more than 0 (varying from 0.330 to 0.925), indicating their hydrophobic feature. In fact, multiple alignments showed that all MeAQPs harbor six TMs (Additional file 6). The average *p*I value is about 8.35, 5.88, 7.98, 7.38, or 9.42 for subfamilies PIP, TIP, NIP, XIP, and SIP, respectively (Table 4).

**Table 4:** Structural features of MeAQPs

Name	AA	MW (kDa)	*p*I	GRAVY	Ar/R selectivity filter				NPA motifs		Froger’s Position				
					H2	H5	LE1	LE2	Loop B	Loop E	P1	P2	P3	P4	P5
MePIP1;1	287	30.62	8.84	0.409	F	H	T	R	NPA	NPA	E	S	A	F	W
MePIP1;2	287	30.74	9.00	0.404	F	H	T	R	NPA	NPA	E	S	A	F	W
MePIP1;3	287	30.74	8.59	0.354	F	H	T	R	NPA	NPA	E	S	A	F	W
MePIP1;4	287	30.79	8.59	0.330	F	H	T	R	NPA	NPA	E	S	A	F	W
MePIP2;1	288	30.59	8.20	0.461	F	H	T	R	NPA	NPA	Q	S	A	F	W
MePIP2;2	288	30.71	6.99	0.438	F	H	T	R	NPA	NPA	Q	S	A	F	W
MePIP2;3	285	30.39	8.89	0.441	F	H	T	R	NPA	NPA	Q	S	A	F	W
MePIP2;4	286	30.42	9.08	0.480	F	H	T	R	NPA	NPA	Q	S	A	F	W
MePIP2;5	286	30.58	8.50	0.449	F	H	T	R	NPA	NPA	Q	S	A	F	W
MePIP2;6	286	30.55	6.99	0.451	F	H	T	R	NPA	NPA	Q	S	A	F	W
MePIP2;7	280	29.76	8.97	0.528	F	H	T	R	NPA	NPA	M	S	A	F	W
MePIP2;8	280	29.53	9.13	0.535	F	H	T	R	NPA	NPA	M	S	A	F	W
MePIP2;9	283	30.04	8.67	0.507	F	H	T	R	NPA	NPA	M	S	A	F	W
MePIP2;10	281	29.93	6.51	0.484	F	H	T	R	NPA	NPA	M	S	A	F	W
MeTIP1;1	252	25.97	5.55	0.730	H	I	A	V	NPA	NPA	T	S	A	Y	W
MeTIP1;2	252	25.97	6.12	0.742	H	I	A	V	NPA	NPA	T	S	A	Y	W
MeTIP1;3	252	25.85	5.19	0.825	H	I	A	V	NPA	NPA	T	S	A	Y	W
MeTIP1;4	252	25.83	5.13	0.801	H	I	A	V	NPA	NPA	T	S	A	Y	W
MeTIP1;5	252	25.69	4.79	0.846	H	I	A	V	NPA	NPA	T	S	A	Y	W
MeTIP1;6	252	25.66	4.94	0.893	H	I	A	V	NPA	NPA	T	S	A	Y	W
MeTIP2;1	248	25.14	6.15	0.925	H	I	G	R	NPA	NPA	T	S	A	Y	W
MeTIP2;2	250	25.41	5.09	0.873	H	I	G	R	NPA	NPA	T	S	A	Y	W
MeTIP2;3	250	25.26	5.66	0.916	H	I	G	R	NPA	NPA	T	S	A	Y	W
MeTIP3;1	258	27.45	7.14	0.567	H	I	A	R	NPA	NPA	T	A	A	Y	W
MeTIP3;2	258	27.32	6.75	0.640	H	I	A	R	NPA	NPA	T	A	A	Y	W
MeTIP4;1	247	25.79	6.12	0.835	H	I	A	R	NPA	NPA	T	A	A	Y	W
MeTIP5;1	252	25.92	7.79	0.749	N	V	G	C	NPA	NPA	T	A	A	Y	W
MeNIP1;1	279	29.94	8.87	0.427	W	V	A	R	NPA	NPA	F	S	A	Y	I
MeNIP1;2	285	30.52	8.90	0.448	W	V	A	R	NPA	NPA	F	S	A	Y	I
MeNIP2;1	288	30.40	9.37	0.430	G	S	G	R	NPA	NPA	L	T	A	Y	L
MeNIP3;1	283	30.46	8.43	0.478	W	A	A	R	NPA	NPA	F	S	A	Y	I
MeNIP3;2	273	28.87	4.96	0.639	W	M	A	R	NPA	NPA	F	S	A	Y	V
MeNIP3;3	274	29.31	6.51	0.659	W	I	A	R	NPA	NPA	L	S	A	Y	I
MeNIP5;1	298	31.03	8.64	0.388	A	I	G	R	NPS	NPV	F	T	A	Y	L
MeNIP6;1	306	31.59	7.71	0.344	T	I	A	R	NPS	NPV	F	T	A	Y	L
MeNIP7;1	299	31.86	8.45	0.551	A	V	G	R	NPA	NPA	Y	S	A	Y	I
MeXIP1;1	294	31.83	6.29	0.707	V	I	V	R	SPV	NPA	M	C	A	F	W
MeXIP2;1	304	32.04	8.60	0.634	I	T	V	R	NPV	NPA	V	C	A	F	W
MeXIP3;1	304	32.30	8.25	0.741	V	T	A	R	NPL	NPA	V	C	A	F	W
MeXIP3;2	307	32.64	6.37	0.788	V	T	A	R	NPL	NPA	V	C	A	F	W
MeSIP1;1	239	26.07	9.17	0.819	V	V	P	N	NPT	NPA	I	A	A	Y	W
MeSIP2;1	237	26.10	9.67	0.479	S	Q	G	S	NPL	NPA	F	V	A	Y	W

*(AA* amino acid, *ar/R* aromatic/arginine, *GRAVY* grand average of hydropathicity, *p*I isoelectric point, *kDa* kilodalton, *MW* molecular weight, *NPA* Asn-Pro-Ala)

Conserved residues, typical of dual NPA motifs, ar/R filter, and five Froger’s positions, were also identified as shown in Table 4. Two NPA motifs are usually conserved, though several variants were also observed: NPS and NPV for two NPA motifs of MeNIP5;1 and MeNIP6;1; NPT or NPL for the first NPA motif of MeSIP1;1 and MeSIP2;1 respectively; and, SPV, NPV or NPL for the first NPA motif of MeXIP1;1, MeXIP2;1, and MeXIP3;1/− 3;2, respectively (Table 4). Most MeAQPs exhibit AqpZ-like Froger’s residues that favor the permeability of water [38]. By contrast, NIP subfamily members as well as MeSIP2;1 feature mixed key residues of GlpF for P1/P5 and AqpZ for P2–P4 (Table 4). Actually, the glycerol permeability of NIPs has been well established [44, 45]. Interestingly, NtAQP1, a PIP1 group member from *Nicotiana tabacum*, was also shown to transport glycerol [46]. All MePIPs represent the F-H-T-R ar/R filter as observed in the pure water channel AqpZ, suggesting their putatively water permeability (Table 4). The high water permeability of plant PIP2s has widely described, however, PIP1s exhibit no or extremely low water permeability when expressed in *Xenopus laevis* oocytes [47–52]. Moreover, PIPs were also shown to transport urea, boric acid, CO_2_, and H_2_O_2_ [45, 53–57]. In addition to water, TIPs were also proven to transport glycerol, urea, boric acid, NH_3_, and H_2_O_2_ [45, 54, 55, 58–60]. NIPs have reported to transport water, glycerol, urea, boric acid, silicic acid, NH_3_, and H_2_O_2_ [54, 55, 61–63], whereas XIPs have reported to transport water, glycerol, urea, boric acid, and H_2_O_2_ [64–67].

**Fig. 3:**
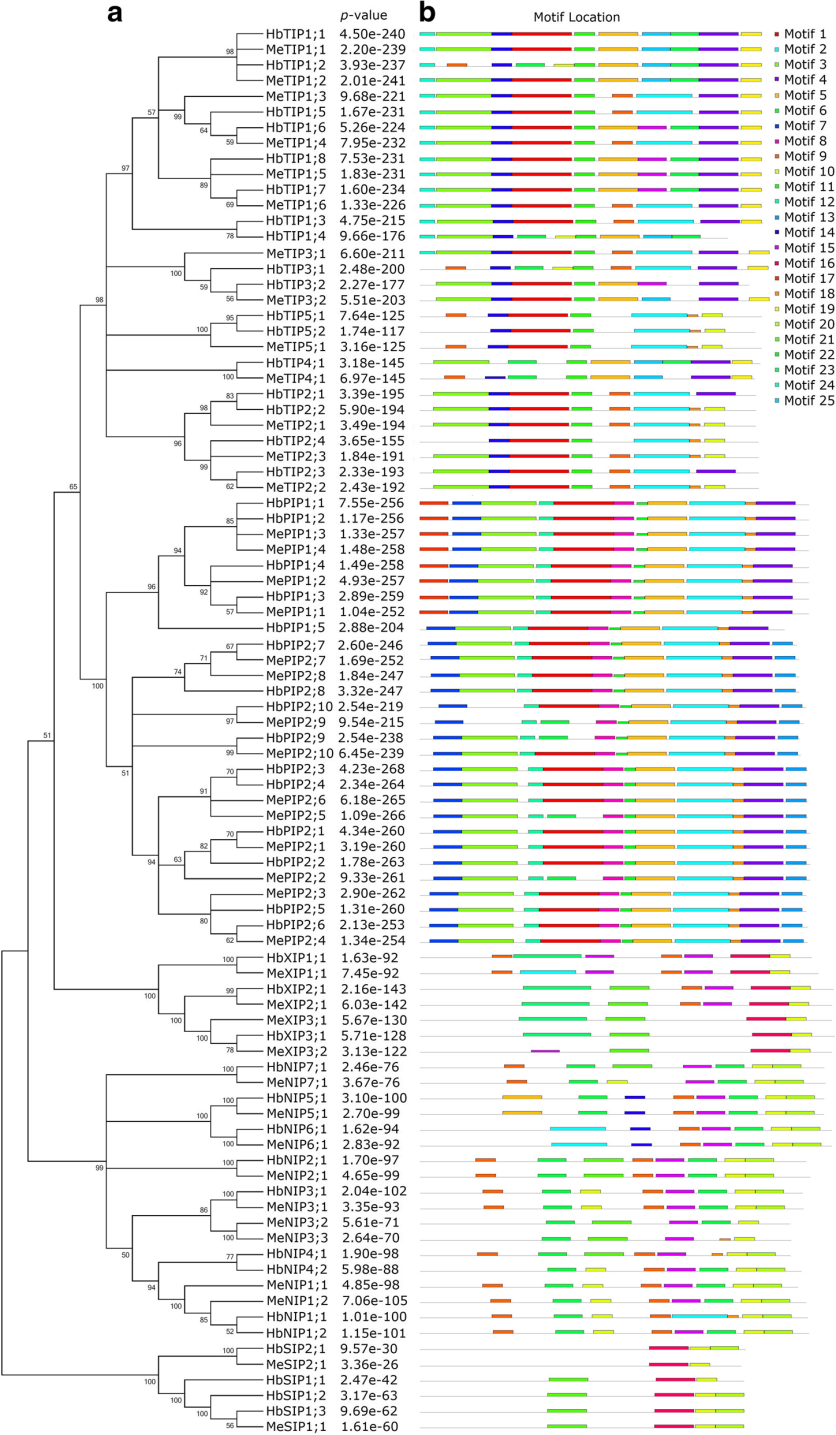
Structural and phylogenetic analyses of cassava and rubber AQPs. **a** Shown is the unrooted phylogenetic tree resulting from full-length AQPs with MEGA 6.0. **b** Shown is the distribution of conserved motifs among AQPs, where different motifs are represented by different color blocks as indicated at the bottom of the figure and the same color block in different proteins indicates a certain motif

To learn more about the diversity of motif compositions among different MeAQPs as well as HbAQPs, an unrooted phylogenetic tree was constructed and conserved motifs were predicted using MEME. Among 25 motifs identified, Motifs 1, 2, 3, 4, and 5 are widely found in PIP and TIP subfamilies; Motif 6 is widely present in TIP and NIP subfamilies and several members of the PIP subfamily; Motif 9 is widely present in TIP, NIP, and XIP subfamilies; Motif 10 is widely present in NIP, XIP, and SIP subfamilies and several members of the TIP subfamily; Motif 15 is widely present in NIP and XIP subfamilies and several members of the TIP subfamily; Motif 16 is widely present in PIP, XIP, and SIP subfamilies; Motif 20 is widely present in NIP and SIP subfamilies; Motif 21 is widely present in XIP and SIP subfamilies and several members of the NIP subfamily; Motifs 7, 8, 12, and 22 are widely found in the PIP subfamily; Motifs 11, 14, and 19 are widely found in the TIP subfamily; Motif 23 is limited to the XIP subfamily; Motifs 13 and 17 are limited to the PIP2 or PIP1 group respectively; Motif 24 is limited to the TIP1 group; Motif 18 is present in several members of TIP and NIP subfamilies; and, Motif 25 is only present in several members of the TIP subfamily (see Fig. 3 and Additional file 7). Among them, Motif 1 spans TM2, HB, and TM3; Motif 23 spans TM2 and HB; Motifs 3 and 23 span TM1; Motifs 12 and 14 span TM2; Motifs 8, 11, and 21 span TM3; Motifs 5 and 9 span TM4; Motifs 2, 15, and 25 span TM5; Motifs 6, 16, and 18 span HE; Motifs 4, 10, and 20 span TM6; and, Motif 13 includes a putative phosphorylation site corresponding to S274 in SoPIP2;1 [20].

Gain or loss of certain motifs was observed within a high number of orthologous groups. Motif 17, which is PIP1-specific, is absent from HbPIP1;5. The PIP2 group usually features 11 motifs, i.e. Motifs 7, 3, 12, 1, 8, 22, 5, 2, 18, 4, and 13, however, Motif 1 is absent from MePIP2;2, MePIP2;5, MePIP2;9, and HbPIP2;9, whereas Motif 3 is absent from MePIP2;9 and HbPIP2;10. Moreover, Motifs 1 and 3, which is widely distributed in OG2-1a, are placed by Motifs 6 and 10, or Motif 9 in HbTIP1;2, respectively. Motifs 2 and 9 found in OG2-1c are placed by Motifs 6 and 15, or Motif 5 in HbTIP1;6, respectively. Motifs 5 and 6/15 found in OG2-1d are placed by Motif 9, or Motif 2 in MeTIP1;6, respectively. In OG2-1b, the motif compositions of HbTIP1;3 is similar to those in OG2-1c, in contrast, Motifs 4 and 9 are absent from HbTIP1;4; Motif 1 is placed by Motifs 6 and 10; Motif 9 is placed by Motif 5; and, Motif 2 is placed by Motifs 6 and 25. Motif 24 found in the TIP1 group is also present in MeTIP3;1 but absent from other TIP3 members. Moreover, Motifs 1 and 3 are placed by Motifs 6 and 10, or Motif 9, respectively. Compared with MeTIP3;2, Motif 19 is absent from HbTIP3;2, and Motif 25 is placed by Motif 15. Motif 9 found in OG2–5 is absent from HbTIP5;2. Compared with HbTIP4;1, the Motif 6 behind Motif 25 is absent from MeTIP4;1, and Motif 3 is placed by Motifs 9 and 14. Motifs 10 and 18 found in OG2-2a are placed by Motif 4 in HbTIP2;1. Motifs 3 and 9 found in the TIP2 group are absent from HbTIP2;4, whereas Motifs 10 and 18 are placed by Motif 4 in HbTIP2;3. Compared with HbNIP7;1, Motif 21 is placed by Motif 10 in MeNIP7;1. Motifs 9 and 20 found in OG3-3a of the NIP3 group are absent from OG3-3b. Moreover, Motif 6 found in this group is placed by Motif 18 in MeNIP3;3. Compared with HbNIP4;1, Motif 9 at N-terminus is absent from HbNIP4;2, and Motifs 18 and 21 are placed by Motifs 6 and 10, respectively. Motifs 15 and 6 found in OG3–1 are placed by Motifs 2 and 18, respectively. Motif 23 found in the XIP subfamily is placed by Motif 15 in MeXIP3;2. Motif 20 found in the SIP subfamily is absent from HbSIP1;1 and MeSIP2;1, which belong to the SIP1 or SIP2 group, respectively (Fig. 3).

### Tissue-specific transcriptional profiling of *MeAQP* genes

**Fig. 4:**
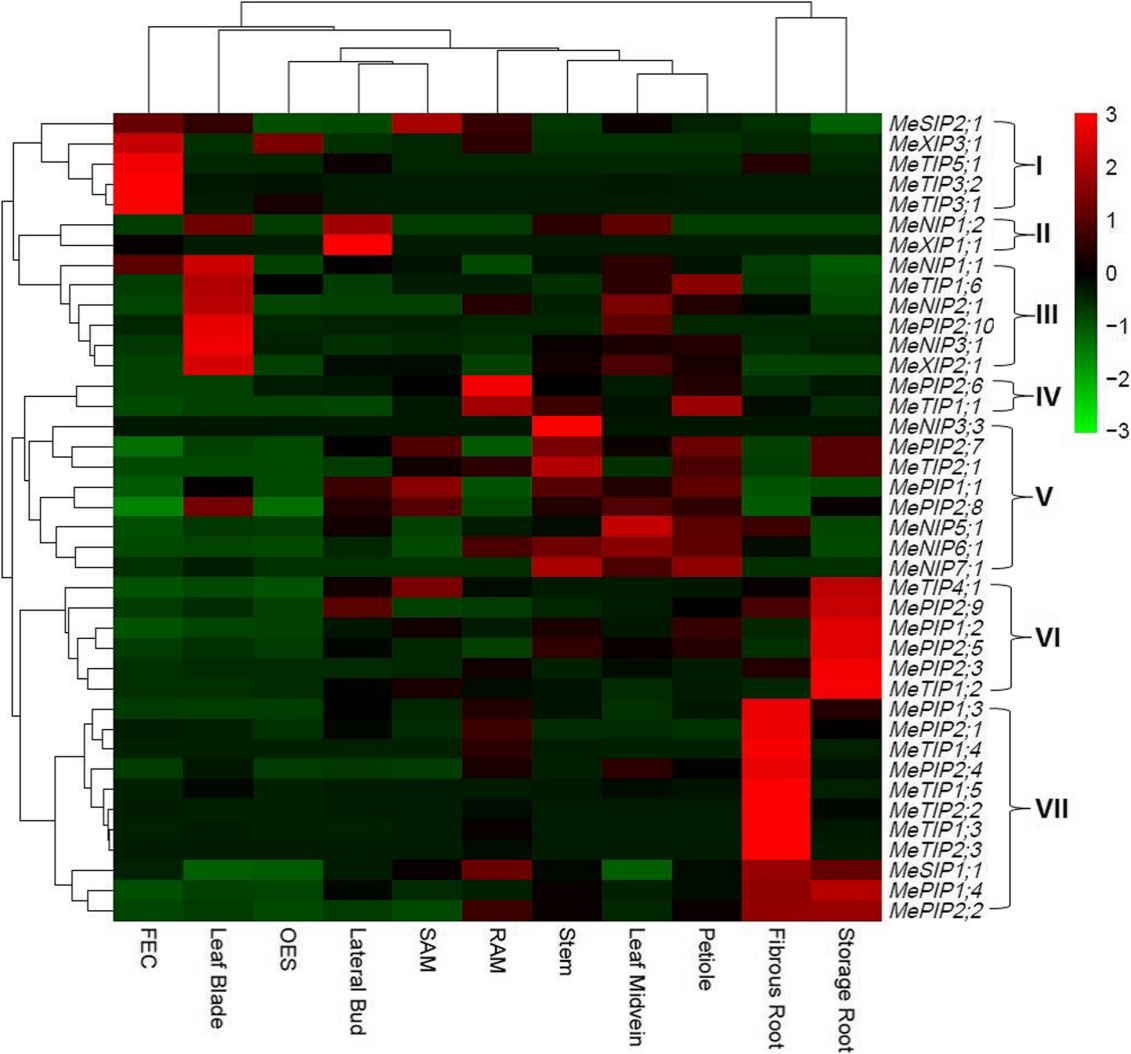
Tissue-specific expression profiles of *MeAQP* genes. Color scale represents FPKM normalized log_10_ transformed counts where green indicates low expression and red indicates high expression

To reveal the expression evolution of *MeAQP* genes, their expression profiles were investigated based on Illumina RNA-seq data representing 11 tissue types, i.e. SAM, lateral bud, leaf blade, leaf midvein, petiole, stem, fibrous root, storage root, RAM, FEC, and OES. Except for *MeXIP3;2*, the expression of other *MeAQP* genes was all detected in at least one of the examined tissues, though the transcript level is diverse. Based on the FPKM value, the transcript of the total gene family was shown to be most abundant in storage root (defined as Class I); moderate in fibrous root, petiole, stem, RAM, and SAM (Class II, accounting for 38.4–66.0% of Class I); and, relatively low in leaf midvein, lateral bud, leaf blade, OES, and FEC (Class III, accounting for 5.8–28.9% of Class I). Subfamilies PIP and TIP contribute the major transcripts in most examined tissues, varying from 83.2% in leaf midvein to 99.1% in storage root. However, the transcript level of the SIP subfamily is comparative to that of the PIP subfamily in FEC, and the XIP subfamily contributes more than the TIP subfamily in leaf blade. Several key members were identified in a certain tissue: *MeTIP1;2* represents the most expressed gene in storage root, lateral bud, and SAM; *MePIP1;2* represents the most expressed gene in leaf midvein, and the second most expressed gene in fibrous root, petiole, lateral bud, and OES; *MePIP2;4* represents the most expressed gene in fibrous root; *MeTIP1;1* represents the most expressed gene in petiole, RAM, and OES, and the second most expressed gene in stem, leaf midvein, and FEC; *MeTIP2;1*, *MeXIP2;1* or *MeTIP3;1* represents the most expressed gene in stem, leaf blade and FEC, respectively. According to their expression patterns over various tissues, 41 *MeAQP* genes were grouped into seven main clusters: Clusters I, II, III, IV and VI are predominantly expressed in FEC, lateral bud, leaf blade, RAM or storage root, respectively; Cluster V is preferentially expressed in petiole and stem, including *MeNIP3;3*, *MePIP2;7*, *MeTIP2;1*, *MePIP1;1*, *MePIP2;8*, *MeNIP5;1*, *MeNIP6;1*, and *MeNIP7;1*, where the latter five were also highly expressed in leaf midvein; and, Cluster VII is typically expressed in fibrous root, including *MePIP1;3*, *MePIP2;1*, *MeTIP1;4*, *MePIP2;4*, *MeTIP1;5*, *MeTIP2;2*, *MeTIP1;3*, *MeTIP2;3*, *MeSIP1;1*, *MePIP1;4*, and *MePIP2;2*, where the latter three were also highly expressed in storage root (Fig. 4).

Expression divergence of paralogs was also observed. For example, the expression of *MeXIP3;2* was not detected in all tissues examined, whereas *MeXIP3;1* was expressed in FEC, OES, SAM, RAM, and fibrous root. *MeNIP3;2* and *MeNIP3;3* were only lowly expressed in stem or OES, respectively. Three genes (i.e. *MePIP1;2*, *MeTIP1;5*, and *MeNIP1;1*) were shown to express more than their paralogs (i.e. *MePIP1;1*, *MeTIP1;6*, and *MeNIP1;2*, respectively) in all examined tissues, whereas *MePIP1;4* was expressed more than *MePIP1;3* in most tissues with the exception of FEC. By contrast, other duplicate pairs seem to complementally express in different tissues (Fig. 4).

## Discussion

Polyploidy or WGD, multiplication of the whole genome content, is an important evolutionary force that acts as a major mechanism for acquiring new genes. Increasing sequenced genomes showed that WGD is widespread and more than 30 events have been described in plant lineages [68, 69]. At approximately 117 Mya, all core eudicots, including arabidopsis, poplar, physic nut, castor, rubber, and cassava, experienced the γ event [27]. After that, physic nut and castor didn’t undergo any additional WGD [3, 4, 17, 70–72], whereas arabidopsis, poplar, rubber, and cassava experienced one or two recent WGDs [1, 2, 5, 6, 9, 11, 12, 29, 30]. Up to date, genome-wide analysis of the *AQP* gene family has been reported in most of these species with the exception of cassava. The physic nut genome was shown to encode 32 *AQP* genes that include one pseudogene (i.e. *JcPIP1;3*), accounting for about 0.12% of total protein-coding genes [1, 18]. Compared with physic nut, castor encodes relatively more *AQP* genes (i.e. 37, also accounting for 0.12% of total predicted genes), reflecting more protein-coding genes present in this species (i.e. 31,221 vs 27,172). Few recent duplicates were identified in physic nut and castor, i.e. *JcSIP1;2*/*− 1;3*, *RcPIP1;2*/*− 1;3*, *RcSIP1;1*/*− 1;2*, *RcXIP1;1*/*− 1;4*/*− 1;2*/*− 1;3* (Additional file 8). They all resulted from local duplication, which is consistent with no recent WGD occurred in these two species [1, 17, 23, 72]. By contrast, except for arabidopsis that experienced chromosomal rearrangement and massive gene loss after WGDs [73, 74], considerably more *AQP* genes were found in poplar and rubber [22–24]. In arabidopsis, 35 *AQP* genes were found and duplicates were shown to result from different modes of gene duplication, i.e., γ WGD (1), β WGD (2), α WGD (8), tandem (2), and transposed (4) [21, 29]. There are 55 *AQP* genes in poplar and duplicates were derived from tandem duplication (4) and the recent WGD (20) (Additional file 8). In rubber, 51 *AQP* genes were previously described, however, three genes (i.e. *HbXIP1;2*, *HbXIP1;3*, and *HbXIP1;4*) were shown to be pseudogenes. The remaining 48 *AQP* genes are distributed across 46 scaffolds (Additional file 1). Although conserved synteny can be observed between *HbAQP* duplicate pairs, whether they were derived from the ρ WGD or segmental duplications still need to be resolved since 7,453 scaffolds available have not been anchored to 18 chromosomes yet [15].

In this study, we present a genome-wide survey and characterization of *AQP* family genes in cassava. The family of 42 members is relatively more than that in physic nut (31) and castor (37), but relatively less than that in rubber (48) and poplar (55) [1, 18, 22–24]. Phylogenetic analysis of 213 AQPs from cassava, rubber, castor, physic nut, and poplar revealed five main clades representing five subfamilies, i.e. PIP with two groups, TIP with five groups, NIP with seven groups, XIP with three groups, and SIP with two groups. The classification was supported by exon-intron structures, conserved motifs, and BRH-based sequence comparison.

Compared with other examined species, species-specific gene expansion or loss was observed in cassava. Except for *MeXIP3;1*/*− 3;2* that resulted from tandem duplication, synteny analysis revealed that other 13 recent duplicates were derived from the ρ WGD shared by rubber. Conserved synteny was also observed between cassava and rubber *AQP* genes, and 39 *HbAQP* genes could be anchored to 13 cassava chromosomes on the basis of synteny analysis (Additional file 9). These results also supported that all 16 *HbAQP* duplicates were derived from the ρ WGD. Among them, the orthologs of 11 duplicated *HbAQP* genes were also preserved in cassava (Table 3 and Additional file 9). Nevertheless, the average Ks value of *AQP* duplicate pairs in rubber (i.e. 0.2609) is relatively smaller than that in cassava (i.e. 0.3730), suggesting a relatively lower rate of evolution of *HbAQP* genes (Table 2). The result is consistent with a slow genome evolution in long-lived woody perennials as well as a considerably longer generation of rubber than cassava [2, 11, 12, 75, 76]. In fact, similar Ks value (i.e. 0.2713) was also observed in poplar, another big tree species (Additional file 8). The Ks value of *AQP* duplicates resulted from recent WGD varies from 0.2442 to 0.5756 in cassava, from 0.1405 to 0.3491 in rubber, or from 0.2011 to 0.5224 in poplar (Table 2 and Additional file 8), suggesting that the evolution rate is distinct between different duplicate pairs.

Orthology defines genes in different organisms that evolved from a common ancestral gene via speciation. Normally, orthologs retain the same function in the course of evolution [77]. Thereby, identification of orthologs or orthologous groups is useful for functional inference, comparative genomics, and studies on gene/protein evolution [78]. When using poplar as an out-group, the BRH-based sequence comparison revealed 34 OGs present in the common ancestor of Euphorbiaceous plants and each group contains one to six OGs, i.e. PIP1 (4), PIP2 (6), TIP1 (4), TIP2 (2), TIP3 (1), TIP4 (1), TIP5 (1), NIP1 (1), NIP2 (1), NIP3 (2), NIP4 (2), NIP5 (1), NIP6 (1), NIP7 (1), XIP1 (1), XIP2 (1), XIP3 (1), SIP1 (2), and SIP2 (1). Interestingly, six OGs are absent from cassava, i.e. OG1-1c, OG1-1d, OG2-1b, OG3-4a, OG3-4b, and OG5-1b (Table 3). Among them, OG3-4a and OG3-4b belong to the NIP4 group which is widely distributed in most examined species, however, species-specific gene loss of this whole group was observed in cassava. In fact, OG3-4a has also been lost in physic nut [1, 18]. OG1-1c and OG1-1d belong to the PIP1 group which includes four OGs. OG1-1c, which is present in both castor and poplar, has expanded in poplar via WGD, but has been lost in cassava as well as rubber and physic nut. The widely distributed OG1-1d has been lost in cassava as well as poplar. OG2-1b belongs to the TIP1 group which also includes other three OGs. In contrast to most OGs in this group have expanded along with recent WGD, species-specific loss of OG2-1b occurred in cassava after its divergence with rubber. OG5-1b, which belongs to the SIP1 group with two OGs, has expanded in most examined species via WGD or tandem duplication, but has been lost in cassava. Moreover, orthologs of *HbTIP2;2*, *HbTIP5;2*, and *HbSIP1;1* have also been lost in cassava after its divergence with rubber. Species-specific gene loss was also observed in rubber, which includes OG1-1c and OG3-3b. OG3-3b, which belongs to the NIP3 group and has expanded in cassava via WGD, has been lost in rubber as well as castor and poplar (Table 3). The loss of OG3-3b in rubber is more likely to occur after its divergence with cassava. By contrast, it’s not easy to determine when the loss of OG1-1c occurred, since it is absent from both rubber and cassava. The exon-intron structure was shown to be highly conserved within orthologous groups and even within phylogenetic groups, though *RcPIP2;5* (a member of OG1-2f) has gain one small intron close to the 5′-terminal [23]. Based on analysis of conserved protein motifs among different Me/HbAQPs, gain or loss of certain motifs was observed within orthologous groups (Fig. 3), suggesting possible functional divergence of cassava and rubber duplicates.

In addition to structural divergence, expression divergence also plays a role in the evolution of duplicates [11, 12, 79, 80]. In our previous studies, tissue-specific expression profiles of castor, physic nut, and rubber *AQP* genes were investigated based on paired-end RNA-seq data generated via the Illumina platform, which revealed similar expression pattern of orthologs in a certain tissue [1, 23, 24]. This case is prevailing between castor and physic nut which usually have no paralog, by contrast, expression divergence of rubber paralogs was frequently observed [1, 18]. As shown in Fig. 4, similar results were also observed in cassava. For example, the transcript level of *MePIP1;4* (the ortholog of *HbPIP1;1*) was relatively higher than *MePIP1;3* (the ortholog of *HbPIP1;2*) in most examined tissues. Nevertheless, different evolutionary patterns were also observed. The transcript level of *MePIP1;2* was relatively higher than *MePIP1;1* in all examined tissues, in contrast, their orthologs in rubber (i.e. *HbPIP1;3* or *HbPIP1;4*, respectively) were shown to exhibit similar expression profiles in bark. Compared with tissue-specific expression of *MePIP2;3* and *MePIP2;4*, *HbPIP2;5*, and *HbPIP2;6* exhibited similar expression patterns in leaf and bark [1, 18, 24]. Thereby, further functional analysis of species-specific isoforms in cassava and rubber is of particular interest.

## Conclusions

This study presents a genome-wide analysis of the *AQP* gene family in cassava, an Euphorbiaceous plant of economic importance. Despite sharing the ρ WGD, 42 AQP family genes in cassava is relatively less than 48 in rubber. These *MeAQP* genes are distributed across 15 chromosomes and conserved synteny can be observed between cassava and rubber *AQP* genes. Phylogenetic and BRH-based sequence analyses further assigned *MeAQP* genes into five subfamilies or 28 out of 34 identified OGs: each subfamily contains two to six phylogenetic groups, and each group includes one to six OGs. In contrast to a predominant role of the ρ WGD on family expansion in rubber, cassava *AQP* duplicates were derived from the ρ WGD as well as local duplication. Compared with rubber and other Euphorbiaceous plants, species-specific gene expansion or loss was observed in cassava, which includes the loss of the entire NIP4 group. Furthermore, gene structures, sequence characteristics, and expression profiles of *MeAQP* genes were also investigated, which provides insights into the evolution of *Me/HbAQP* genes, especially functional divergence of recent duplicates. These findings will not only improve our knowledge on family evolution in Euphorbiaceae, but also provide valuable information for future functional analysis of *AQP* genes in cassava and rubber.

## Additional files

Additional file 1: Accession numbers of AQPs identified in rubber, castor, physic nut, and poplar. (XLSX 130 kb)

Additional file 2: The gene model for *MeSIP2;1*. (PDF 177 kb)

Additional file 3: The gene model for *MeNIP1;1*. (PDF 78 kb)

Additional file 4: The gene model for *MeXIP3;1*. (PDF 96 kb)

Additional file 5: The gene model for *MeXIP3;2*. (PDF 100 kb)

Additional file 6: Alignment of cassava AQPs with structure determined Spinach PIP2;1. (PDF 237 kb)

Additional file 7: Detailed information of 25 motifs identified in this study. (JPG 409 kb)

Additional file 8: List of recent *AQP* duplicates identified in rubber, castor, physic nut, and poplar. Ks and Ka were calculated using PAML. (XLSX 12 kb)

Additional file 9: Matched positions of 39 *HbAQP* genes on cassava chromosomes. The positions were based on synteny analysis, where *HbAQP* genes were marked in orange just following their syntenic genes in cassava. (JPG 1561 kb)

## Abbreviations

AQP: Aquaporin
ar/R: Aromatic/arginine
BRH: Best reciprocal hits
Chr: Chromosome
EST: Expressed sequence tag
FPKM: Fragments per kilobase of exon per million fragments mapped
GLP: Aquaglyceroporin
GRAVY: Grand average of hydropathicity
Ka: Nonsynonymous substitution rate
Ks: Synonymous substitution rate
MIP: Major intrinsic protein
MW: Molecular weight
Mya: Million years ago
NIP: Nod26-like intrinsic protein
NPA: Asn-Pro-Ala
OG: Orthologous group
ORF: Open reading frame
P1-P5: Residues at P1 to P5 positions
*p*I: Isoelectric point
PIP: Plasma membrane intrinsic protein
RNA-seq: RNA sequencing
SIP: Small basic intrinsic protein
TIP: Tonoplast intrinsic protein
TM: Transmembrane helix
WGD: Whole-genome duplication
XIP: Uncategorized X intrinsic protein
