# Supplementary material for: Genome-wide comparison reveals divergence of cassava and rubber aquaporin family genes after the recent whole-genome duplication
Source: BMC Genomics. 2019 May 15;20:380. doi: 10.1186/s12864-019-5780-4 (PMC6521647; doi:10.1186/s12864-019-5780-4)
Supplement: Supplementary file 2 — The gene model for MeSIP2;1. (PDF 177 kb) [file 12864_2019_5780_MOESM2_ESM.pdf]

Additional file 2: The gene model for *MeSIP2;1*. The coding region is marked with uppercase letters, under which are its deduced amino acids. The transcribed untranslated regions, including 5' UTR, intron and 3' UTR sequences, are marked with lowercase letters. The start and stop codons are marked with bold letters.

1 ggatagtgggtaatagtaataaattgaaaagttttcattacaatccatggtgattaaaca
61 tgtttttttgtggcgtataaaatacaacagaaaaagaacctaacagctcatcctattttg
121 accggctgcgataacaacaggaagatggttttgagaaaaagcggagagcctcacctgcga
181 caaataggaaatagagcaggaagctgtgtggtgctcagtgacaacaccacctcctctccc
241 tttctataataaaaacaacgtccattttcatgcattagttacacaggaaacaaaaaaata
1 M D S A V T L R
301 aaaataaaaaaaaaaaaaaaaaaattcagggtggaa**ATG**GATTCTGCAGTTACTCTTCG
9 L I I S D F V I S F M W V W S G A L I K
361 TTTGATCATATCAGACTTCGTAATTTCTTTCATGTGGGTATGGTCAGGAGCTTTGATAAA
29 I F L N R F L G L G H H E P R D E A I K
421 GATTTTTCTGAATCGTTTTTTAGGTTTGGGGCATCATGAACCCAGAGACGAGGCTATCAA
49 A A F S I I N M F F F A F L G K I T N G
481 AGCTGCTTTTTCTATCATTAACATGTTCTTCTTTGCCTTCTTGGGTAAGATCACCAACGG
69 G A Y N P L T I F S S A I S G D F S Q F
541 AGGAGCTTACAATCCTCTCACAATTTTCTCTTCTGCAATCTCTGGGGATTTCAGTCAATT
89 L L T V G A R I P A Q
601 TCTCCTCACTGTTGGGGCCAGAATCCCTGCTCAGgtttttactctttttttgtttcccttt
661 ttttttatactttatatttttgtttcaatagctgaatatctccgatggagtaatttgga
721 cttgggtttcaaattatttctttttttgttttatgggttgcctgaaatttaatgcacaaaa
781 tttgatcttttcgttttctcagtaaaatgaaaacgtgatttgaagtccagattggttgtg
841 atgtgaataatattctcgtccagtcattttcccctgttgtttctaattttatctcagaat
901 tactggatgtggatacggcttttcatattgtgctagtaatttttctgtagatcttaaatt
961 tttggaaagagttgaagaattcattattatcaacatatttgcatttgtttttctctcttc
1021 ataatactctctttacccacaatttttaagaaacagaaaaaaaaagaaataatttgatgc
1081 aaataattaggttgcccttgaaaaagcagaaagggaaatgtagccaccctcacctagtca
1141 gctttgtttgatgaaattgcaaatttagtgaagaaaatacgagagtcccatatgttagta
1201 gtccctgatttaaaatttgagattcaactgaattattaccaaaatatatattgtgaattt
1261 gatgatatttgtgaaaaatgtcacaactaaattttacattcgtatgaatttagcatgcta
1321 aagataggggtgatgatggtggtaatatggttttcgaatgacaatgatgttatctaacaa
1381 ctataggtgcttttaagtaatggtaatttctattaggtcaatgcagtaatggttttattt
1441 tgggtccttttctaggttcttgttaaggattgaatccataattagttagttacgggtaat
1501 gaaagagaaaggaatagaatcagaggaataagaaagactatgtaatagaaatgagagaaa
1561 atagagtagaaatgatgagatatttattgggatttcttgatattgattcagacaattcaa
1621 tgcaaatatcttttgctgctatttatacagcctgctaactcagctgaaatacaagttctg
1681 gcgggattcctttctttctaactacttgtgttacatttctccaacaactaaacatggttt
1741 gtattacttaagacgattttcaaattctggatccttcttatttggattcttattttcata
1801 tggtctgctaagtccttccagctgttccaactgaaatacttgttctgctgagattccttc
1861 tgttacatttctcagacaactaaaacatgatttggattacttaaaaggattttcaaattc

1921 tgcatccttttcttatttggattcctatttgctgtgcatctctagagagtcaatttcttt
1981 tcaaccaagggggaacgatgcagggttttctagaaattttgggactgcctattgtaggaa
2041 gatatataggaagtagagttttattttatttaggaagaagtatttgtttggtatcgctaa
2101 ttagtatttcttgtttagggttctaaatcctaacagtagtaggattattaattctactat
2161 aaatagtatctactatctacatcctttgggcatttagatagatcttttccatgattggaa
2221 attgatttgaaaataaataaaatttagaatttatatctcttccctttgttttattggttc
2281 taccattctactacctagagggacggaatttccttaaggactgtgtcaaacatgcctcgg
2341 acaagtgcaataactgtgaatatctgctaacactaataacagtaaaaaacagtaagtaca
2401 acctgttgaaaattaaaaatagtcagcagatgtgtgctttcttgaagaggttaagttgtg
2461 aaactcttcaattggtattgagtgttgacttgatgtataaattgagaaatagagtgagtg
2521 ttgacttgatgtataaattgagaaatagagatggaagaaccttatgattaataaattggc
2581 ttaatcaatttacaaattcaatggtctagttcaggacaaagaattacaatatactttttt
2641 tttggcacaagtaatgagaagtaaatggtgtcatattcaaggtcagaagtttatgatagt
2701 gtaacactttaaacctcaatgctgagctttatcagtcttgcagagtttaatgcaaatatg
2761 agaagataaaattccagcaatgattgttttcttattttaatggtcttttattagcatgaa
2821 tattaagtttgaggtacattttgttttcactcaatttccttttaaggttatttgatgttt
2881 tagaaataattggtattagttagaatctttgttagttattatatatctatggtttttttgg
2941 ccttaggaattggtcactcttcctttatgaaaggaactcttgaaagtacatacgttgtct
3001 ccaaatcagaacattacttggagtggggtcaattcttcattaaaaatgaccatgagagta
3061 ttaattctctgtcacacttttttgcactctaacatttttcctttctttttctttgcatcat
3121 ttccttcatttgttcttcatctctaccattattactcttctttatctcggttccactgtt
3181 cattcgtgatgatatttgcagccccataccaaatttgcttaatgcatgtgataagggaat
3241 gagacatgcccatattgagataggagagggcaataactttgcaattccaaaatggccttt
3301 tgtttacgagggatgtgtttatttggactttggagggaggatatgagttaatagagttaa
3361 aagttaatccctttttaatatgtttttgtctcctcccctatcaaggaggggggaaagggtg
3421 agaataaagaaaaaatatttatttaactttttttagtgttgagattctagatattctagcc
3481 ttggtctcctaattagattattgtaatcatatgtgatttcctaattagccaaaaatctat
3541 atattcctatttgggtttgtattcctattctataatcttctgtaagctatataaaattca
3601 tcaaggttgaggaaataatcaagtgctataatttcttcacaatattttctctctttagtc
3661 tttacatggtgacctcctttttctttctctttttaatctctttgctttaagagatgggct
3721 tgccatttcttgttgaaagccttctagaagccaattggtcgcaccaccctcctcgctagt
3781 ctcgttagcgattgataggtgcctcctcaaagtgttgtcgcaagagctagcttcatgcac
3841 agccacacatcgcctcttcttctcaacctccaatacatatgctagtgcatgacgactttc
3901 cagcaaatatttatagtggcgacatcttgagtagcctcgccaatggtgggagaccccaac
3961 caagtgggtcgcatcacttgccatcccttcaagtcttccctcaggtctccaaacacttct
4021 ttttaattttaccaactagatgttaacatgagaagtaagagtcttccaacataggttcta
4081 tgattcttaaacctatttgctcttcaaatgcaagctctccaaatattactactatcaaat
4141 atattgagttgcatcagtagaattataatttatggggcaacatatgatgatcatctagaa
4201 aaaattgctttggataccgcttttagcaatcaaacaaattggactaagattgatgtgcaa
4261 ttatgctgtaccttatattatggcactcattacatccaattactagttttattttagtca
4321 tgtaagacgtgtagtaaggtctggcctaagcaaaatattatacaccaatgatgtgcattg
4381 tatttatgaagtagtatcaaacacagttcattcataaaaaaatcattcagatatggctat
4441 ttatttgggacaagtagaacattgaaaaatgaagttaaagctctcatgccttttactaat
4501 agtgtgactgcataagaaaatagcgagacaagttcttcatggttctagcattggttgggc

4561 ttccatttgatcttgttgctataaaagatgaaacactaatttgtttagtttagaggttgt
4621 atttgcaaggctagctctcatcctgcttattaaggatatctcgtcagaaatggaatccta
4681 tccgttggctatatagaagggaaatctacaagaacaaggggctaccaaaaaggtaaaaag
4741 aaaaaaaagtctcattgcatctattgtgataataatgctcacaatcaatatgataatact
4801 ggtagatcaactactcacatgatctacagtaatggaggtggtttgcttccattacctaat
4861 atgtaagatcagttgccacactccgtcatcttaagtggtgcagataacaaggaatattta
4921 cagttccaagcagtaaaataatagtccttttcattcactatttatcacttaagtaattct
4981 tttgcttctctaacaaagtctttacccgttgtcattggtccatggattctagattctgat
5041 gcttctaactgcatatgtagtaataacaacattttctccactcttatttctcctttgacg
5101 ctatttaaagtcaatttagctaatagttccaaaactatagttaaaaggatagaagtaaat
5161 attcttccttctattcctttaactctagtcttatttacccttgaatgctcaaatagtttg
5221 atttgcattagtaaattgactaaaaattttcattattttgtaaatttttttggttgacttt
5281 gttattatgtaagattagatcacagggaagatgattgaagtaggttataagtcacaaagc
5341 atgtctcgttgtgcacttgagagtcaccagttattttcttcttcttcttctattttgctt
5401 tctttcctctaattcttctttcttcttcctaaaatttctttctttcccgttctttcttct
5461 ctcttctttttttcttggcagaatctttcttcttctatccctgggaattcttctccttctt
5521 tcttttccataattttcttcctctcatttgcagaaatttcttctatttccttccttctgt
5581 tcttgctctgtctttctttttcttggcaaaaccttgtttctgcacttctttctttccttt
5641 ggaacctctttctttcttaaattcttgaaccaaattcttttttgttctattgttttctag
5701 ttcttatattttcttctttaattcctcactttcagcatttcaagaacttgtttcttggaa
5761 ttcttgaaattttcgttccttcctttattcacattcttttcattcttcttcttccgactt
5821 tattgatttttccataaaatttctcccttgaattatcatttttacttcttgaaaatcagat
5881 tgctcttcttgatttctttgtttccttgaaatttttgaatcatggaagtgcaatatttg
5941 gcacaagtttttcttaatgaagttcaagaaattctagatagaagataaaaagaacaaaaa
6001 gagtggttggaacaaagatcgaaaaagtgattcaaaaagtagaaaatttagattagaaaa
6061 tggaaaggtttgaacaatcaattcaagtgacaaagaaaaagattgcagtattgatcaatg
6121 aaaattaaatttttgaaaagcatactaaagaacaagatgtctcatgtgaaatttccgata
6181 agaaagacagccattgaggtggagcaggagctttttgttgacatgtctaatgtaagtgag
6241 atcaagaaggagaaaaatgaagatggtgatgatgggcaactaaagtttgaaatgaggaca
6301 atattagttgaatctaaaatgtttgcacaattcagtcccaaaactgagtttttttgctact
6361 gaaattcttgaaaatgttcttgaagaaaagaaagataaaggtgtgggggctggaaacatg
6421 tggcagtatttttggagctaagatggtgagtacaacggttgttgtgacattcagcaccat
6481 ccttgggaaaggatcccacattggcggtttatggaaaatcaaaattgtatatataatagctg
6541 gcattaaactactaaataacttgggttaatcattttgagccaagtggaaagtgggtccaa
6601 aagttatttgggtcagtttgggttgggctgtttcaattggtatcagagccaccctggatc
6661 agaaaaattgtgcagagctagtgggcctgggaggaaaaggctgcccgtgtgagacgaggt
6721 ggaatcgccccgaggtactagcgaaccacaatacccgagggttcggtcctaacttagcaaa
6781 ctattttggaccaagtggaaagtgagtccaaaagttatttggatcagtttgggttgggtt
6841 gtttcagttgtcatcatgtaatccttatggaagataaatgggaaataacaagaagagaaa
6901 ggaaaaaggttatgacatcaaattctattgtaatcttgaaggaacaagcatgggaaattt
6961 ggagaaggaaaaggaagaaaagtttagcatctttagacttcagctaagctttatgtgtcat
7021 ttcagctgaagaagaaaaaggggaaaaaaaagttcctcgtcaataaagagtccataaaat
7081 cactcacaactttagcccaactttctagaacatttcagctgaagcatgtgctggaagtta
7141 acatttggggttctaaattcaggggattaattgactagcaaaagcttctatgtggcattt

7201 tcacatccttctgaccaagtctgaggaaagaagcaatagaaattcatttcaattttcaac
7261 attttcttcgtttctaaggacaagaatgatttcaaggggtagggaatgttatgagtaagg
7321 aataaagaagtattggattagtctctgcattcagttgtaacttgtaacggtagaaagtaa
7381 ttgtggcacccaaagtagtataaataggaacttatttgtgaagataaaaatatccaagat
7441 cattagcaattaaagataaactctttctctctacttcttctctttcctcttctctgattc
7501 tctctgttacttctccccttctatttctttttgaagaattttgagaaaatttagagtat
7561 tcttgtatttcactcttaatctttacaattggtctatatgactatttatacacaaagaat
7621 tatatctaaacaggaagcaaataatcaaataataattatagagataattaaggatcccaa
7681 tcaaatcaaatcatatctctagaatcagttaggattagcaaataagtcaacactccctct
7741 caagttggtgcaaaaatgtcacacatgcccaacttgcaaatcagattatgatagatcttg
7801 ttgttgagtcctttagtaaacacatccgcaagttgcccagtagaagtcacatgaactagg
7861 ctcaagacactgtctattaatttttctttaatgaagtgccgattaatttcaatgtgcttc
7921 gtctagtcatgttggactggattttgtactatacttgtagtagttttattgtcacaatac
7981 aaagttatcttgtctctctcgagcaacctcaactcctccaataacttctgtaaccataag
8041 agttcacacacaccttgggccattgctctatattctgcttcagcacttgaccgagcaaca
8101 acactttgtttttacttttccaggtgataaggttctctctcacaattgtgcagtagtca
8161 gaggtgaatctcctgtcatcaagagatcctgcccaatctgcatctgtaaaagcttcaact
8221 cggaggtgaccatgtttggagtaaagaagccctttccctagtgcagactttaggtatctt
8281 aagatgtgaagtacagcctgcatatgagtttcgcgagggtcatgtatgaattggctgact
8341 aagctaacagcataggctatatccggtcgagtgtgtgagagataaatcaactttcctacc
8401 aatctctggtatcttttaatatccacggattcaccagttcctgcttccagcttgtgatta
8461 ctttcaatgggagattctgctggtttacaccccattataccagtttcttccaaaagatcc
8521 agaatgtactttttttgggagatgaaaattctttttctgatctagtcacctcgatacct
8581 aggaaatattgcagttttcctagatctttgatttcaaattcctaagtaacctctttggtt
8641 gagccatttcctctttgtcattgctggtcaccattatatcatcaatataaacaataagaa
8701 gggtgatcttacccttgtgatgtttgataaacagagtgtgattagcattactttgttgat
8761 aaccaaaggacatcatggctctgctgaacctgtcaaaccaagctctgggagattgtttta
8821 gtccatacaaagcctttttaacttgcacacctttccttgtgtcttctcatcagcgaacc
8881 caggaggaatttctatgaacactttttctctaaatctccatggaggaaagcattcttca
8941 catcaaaccgttacaagtcccagtccaagttggctgtgcaggataacagaactctaattg
9001 agttcatttttgcaactggggcaaatgtctcttggtaatccactgcataggtttgggtga
9061 atcctttagcaatcaacctggccttaaaccgttcaattgtgccatcaactttgtgtttca
9121 ctgtgaacacccatttacagccaatgagtttttttcccaggtgagagagtgacaagctccc
9181 atgtctcatttttagccaatactttcatctcttcaatcattgctcccttccatttaggat
9241 ctgcaagagctttcttccaatcctgtggaataggcacagaggaaatagacaaggcaaagg
9301 ctctataggaaggagacaaagattcataagaaacaaagttaaaaatagggtgtttagtac
9361 aatatctgacccatttctttgtgcaataggtttatctaagtcattggagcatttaagag
9421 ttgtgggtaactcactagaagaggattcttgactctgagtagactgcataatggcttttt
9481 ctgtcttgtctctcctcgaatatcttctcaaatctggcttgtccaaacgacaaattctct
9541 ctccttgattggacatctccccctgtctactagaactcaaactatctagttgaaccatat
9601 cattcagaatcacagaattcgggatcacctcttcttgctcattattctcctcctgaagaa
9661 gtgagtggatggaactgaagtagggttcagtttctcggaaggtaacatccatactgacta
9721 agtatttcctagagggaggataataacacttgtatcctttttgtgttggagaatacccaa
9781 caaacacacatttaagggccttgagatccaatttgcctgtcgtccagtatggacaaagca

9841 aacacacccaaatacttttggtggaacaatgtatgaattcttcctttgtagaaccgccaa
9901 aggactcataaagtcaagagtcttgagagacattctattgataagataagcagatgcaag
9961 gttgcatcttcccaatatgctttgggtagattcatggtaaacataagagatcgagctacc
10021 tctaatagatgtctatttttcctctcagcaatgccattttgagcactagtataaggacaa
10081 ctagtctagtatactatcccattactctccaaataagtagaaaagctactatctatgtat
10141 tctcttccattgtcagttctcaaaattttcaccttagtatcaaattgagtacaaaccatc
10201 ttatgaaaagattgaaaacaagaaaagacatcacttttagctttaatcaaatagacccaa
10261 gtcattcgagtgcaacaatcaataaaggtgacaaaccatcgattaccagacaagaagaca
10321 gtttgagtaggcccccaaacatcagaatgaatagtcgcaaaaggaatttgacttttatta
10381 tgactcaaaagataggatgttctagtgtgtttagcatactcacaagcatcacaaaataga
10441 gaatcaaactgagttctagcaaacaaattaggataaagtttttttaaggcaaaaaatgat
10501 ggatgccctaaccgcctgtgccactgtattatttcctgattgacatccttattttctcca
10561 aaacaggcttgagatatcgaagaacctggatcaccctccaacacatataagccatcatgc
10621 aacctaccattgccaatcctctttcctgtgcgaagatcctgaataacacaatgatcagga
10681 aagaattcaatttttacaattaagggcattggtgatagcactgacagataaaaaattgac
10741 aggaaagtggagaacatgaaggacagatgataatttaatgttggatgtgcaaataacaga
10801 gtctgttccgaatatgggagtaaaagagccattagcaattctgacaatatctttggttag
10861 agaaggagaataattgagaaagtctttagaagagcctgtcatgtgtctattcgcaccaaa
10921 atcgataatccatggaatattattcagagaagaagcattatttgactttacaaagttaga
10981 tgtgacaccggaaaacgagtaatcagagttaggaatcgaaacctttttaccttctgccat
11041 ggtaaagatttatgaaccgtgaaagaataggaggtacctaaggttgtacacacaagagag
11101 cccaatcgattcaccaaaagaccggttagcagcatgggaaagccgaaaagatggttggaa
11161 tcatcgttggagtgatcggagaggcaaagcagcatcaggcgattggtcactcgccgggac
11221 gcgatgggtcatgcgccgggaaggggaggcgcgtaagcctcacgcttgggcttttccggc
11281 gtctgagctgggcgatcggccggcgacagtccgacgattctcctggtgccagcggtgaga
11341 ggaggaaagactcctagacgggttagttctaaaaaaggtaaatctagggtttcaaaaccc
11401 taaagaggaaaaattgaatttgaactctaaaatcaggaaaagctctgataccataaaaaa
11461 ttttgaaaaaatttagagtattcttgtatttcacttttaatctttacaattggtctatat
11521 gactatttatacacaaataattatatctaaacaggaagcaaataataaataataattaca
11581 gagataattaaagattccaatcaaatcaaatcatatccctagaatgagctaggatcagta
11641 aataagtcaacactttctcagccattttacttctctctttctctctgctatttctctat
11701 ttcaaagggaattgcaagtctgccatgtaacaggttgttatacttttgtagtacaattga
11761 attaaccatcatgtactgcatgtagttgaaagaaaattaatataaactttgttgttatta
11821 atgatagttatctatcttctaattttgttatcattaatgtatgatgcgctagatggttga
11881 tattgtttagcatgtctcaattcttttcttctgggttgatattgtttaataggttatcta
11941 ccaatttaataggtgcttgcctgctgctatggtgcagcaggcacctgctgttcacagcag
12001 cagacctgctgctgctaccacagcatcaacaagtgcctgctgctgctgtttgcacagcag
12061 gagggctgctcacagcagcagactgctgtggtgcacagcagacaccacagcagcagcagg
12121 aaactgctgttgacttgctgcccacagcagcagacctgctgctgctgtgcaccacagcag
12181 caagtgcctgctgctgtggttcaatgtttaagctcccatattaaacatccataatgttag
12241 cagattttatttctaatcagtagaataaaacttgaatcaaacaagggctagtttgtagta
12301 tctattgctaaacaaaacaatagatcatctatttaaatttactcccattcggtgactcct
12361 accatcatgtaaaactaatagcaaaatgacaaatcctatctttcaggtcctgtgttcaaa
12421 ctattaaccaaaagaaaaaattatatattcaggctatttttttctttgtcaaaaattcagg

12481 ctacttgagtaatgtttaagagatgatctaaacttggaccaatttaaacgtaggcaatga
12541 ttttctaagtaaattttagtgatgtaccatcatgtaaaactaatagcaaaatgacaaatc
12601 ctatctttcaggtcctgtgttcaaactattaaccaaaagaaaaaattatatattcaggct
12661 attttttctttgtcaaaaattcaggctacttgagtaatgtttaagagatgatctaaactt
12721 ggaccaatttaaacgtaggcaatgattttctaagtaaattttagtgatggaaacaaagct
12781 caccagtttgataaatatggtcaaaatgcattgaaaattaaaatataaggatgtgttatc
12841 attgatataaaggcaaaagccagtaatgctacaatatcattcccagcttaaataaaaaca
12901 actcaaacaataaaataggaactcaaattaaacatggaactcaaattaaacatacttacg
12961 tcttttagtttgtcctaagctgcaccagcatactgcaatcaaaggaggtgaaggaatgaa
13021 tatcaaagaatgcagagaatatagaatcaatgttacaaataagtggattgtatgtataca
13081 taagagcatcagatccaaggcagatccaagccacaagaaataatgataggaatgtggaga
13141 gaagggagaatgtagaattaagaccatgagagtgtagtttgagggtgaactgccaaccct
13201 agtcattgcaccagttattccaaacctaaaaggagcacttttgttttgccaatctgcaac
13261 agaaatagtatgcctcattgaaaactgtcctatatgcaatgcaaatgttcaggcatcata
13321 tacagaataaaaaaatggtcaaaggatatatatgcagaaacttgagcaccaaaattgaaa
13381 tacactagttaatgattctcgtcaaaagacatacagttattttcatgcagttatttgcaa
13441 ggacattaaccttcataaatgaagcaaacaaaagcatacctaagcaacaatgggtaacct
13501 tgaggagaacactacctcagcaatgggccttccaatcttaacaatcctccaatctcggca
13561 gtctagcaattttaatctcagcagtggactcgattttcgtagcaagctagcaggtcccaa
13621 gcaggtatcctcacagtttcgcagcaaccaaatcccaacagcacctctgctcccaaacgc
13681 gcagcaagcatgtcaacctcccctcccaaacgctcagcaagcaggtcccaatctcctcct
13741 aaaatcgcaagaagcagctccaatctcctctcaaaattacaggaagcaggagtaagtgga
13801 agagcaatttcttctttataatgtggccacaaaattctagtttagtcactcctaatctct
13861 gtacaaaataaaactcagtaaaatggagagatagatgatagatgaagaatccccaaaatt
13921 ctgttctggttaaggagaggattgggattaagtcttatgaaggttacctttaggagacta
13981 cctcctgtgggagggggaaaggaaatttgaagaaaaaaactctataaaataaaaaaatgta
14041 tatttttcttaataataatagtgttattgttattttcaaaacacaaaagatccaaactag
14101 gtttaacgatggctattttaatttttaacgataaacattatacgttataaatttatataa
14161 ttacgataagttttatagttggtcgttaattatataattaacgaaagatgtttaatttca
14221 tcgttaatatttcgttgttaaaagccatgttacttgtagtgtttgatatttcggttaaag
14281 ttttatgtattgacaatgcaagggactctcgctttctctttgctcctttttttattttctt
14341 ctcaaggtattacttgctagaattcttgtacaacatgtccacaacaaaatggagttgaaa
14401 aaaaatcagcatttaattgagactttctagaccttatttatatattaaaatgttcctcc
14461 tcctttatggggattttgttctcactacatgctacttgatcaatcacttaccctcttta
14521 ttttgtagcatcaaaatcctcgttctattttattttttcattcaggattcacattagttgc
14581 ccttatgtgtgtttgcgtgtacttgctttattcatgatcatcctcctgaataagacaaac
14641 ttagcctatatcaatcaaatgtgtatttttaaaatttttcttgccatagaaaaggttaaaa
14701 atgataatgactctactgccaacaaatattttgtatccgccgatgcaacctttgaaacat
14761 ctccttacttttcatccaacttaacacatcataagtttattaagtcttgttagttcctac
14821 tcttcctccacctcaaattagccaatctcctaccactgtccaaccgctccatgtctactt
14881 tcatcatcttcccactctcgctaccaccactaatgctttttttagcagagcgaaaagggtt
14941 ttccatgaaatgggaaatggaaactattcaccccatgtgaggtttgtgaataagcaattg
15001 tctaataataagcaaggaatatacatctttttgctaaacagggtgtgttgaactcataat
15061 tcattggatactttttatgaatgtcatttaagtatcgtaaataaattgcttttaggattt

15121 ggtggtcttcttattgggcaaatctactattagatgtcgttgggttttacacaatcaagg
15181 tggatctagatggcaaaatatctatatagagtgatcactaggatcggttgcttggaagtc
15241 tagcttggtatgcacttatgatgatcattatatggcttaaagcagtatccaagaacatgg
15301 ttgaagagatttagtgatgtggtccaataatttagagtgtttcaaagtgaagttgatcat
15361 tcaatattttctttaaatatactaaaagagattagcatgatagattgttgacatgtggat
15421 gcttctatggatctaaatgttaaatttgtttttagatagaggaagccattgaaagatccc
15481 gatagatactcacaatcactcaactaaacatttcctttgctgttagtgtagtttagtcaa
15541 tttttgcaagtgccgtgtagtagtcactaggatgtaattattcacatttctaagtatata
15601 aaaaaaaacccctagataaggcttgttgtgtaaagagaagtgtcattcacaaattgttgg
15661 atattcaaatataggctcagaaagctctcatcagatagatgttctattttaggatattgt
15721 gtcatgtatgggaggaatttaatattttgaaagagtaaaacgcaagatgtggttgccaag
15781 tctagtgcagaagctatggcctcagcatcgtgggaacttatatggaatagacaacttctt
15841 caagagttgaagtatgggaaaagtaagcaaacgaagttgttatgtgacaatcaagttgct
15901 cttcttattgcctctaattcaatctttcatgaaagaacaaaacatataatgtgttgccac
15961 cagtttcaataattccaatgatcagttagtaaacatcatcactaaatcccttaggagttc
16021 taaattgggtacatttctagcaagcttggagcatatatgacctatacgctccagcttgag
16081 aggggtgtcgagattctagccttgatttcctcctaattaaatttctgcaataatttgtaa
16141 cattcttaattagcatagaatctatactcttagcgggcatatattcctatttataatctt
16201 ctgaaagctatataaacccattaaatttgaatcaaaagctctataatttttttgataatat
16261 tattctactctttacaataagcatttgtcactactgaccccaactaattcggaattgaag
16321 agtaattgtagttgttatgttgttgtgactatttaaattaaaataatataataaataaat
16381 agtattaacctttttcttttctaatcctgtatgaaacatagttcttactcgtaacttctgt
16441 gtcatttttttgcttcttaagtttagcacccttatatgttgcttccgtagcttttagcttc
16501 caattcttcaacttcaaaacaaatgcaccagaaatgatactgttacaatttagagagaaa
16561 ttgattggagctctcaggtttatggcgaatgagctaatcattttttgcaatatttttcttg
16621 gaaagaatttgattgttgtgaggcagttcaactttttgctttttttttttttttttaaaaaaa
16681 aaaaaggttctggaaataattgatcatatcattttagtagatacaataagcggatatgag
16741 caagtctgaactgttacacaaaggcaacttatttggcaaatcatctgtccgttgtctttt
100 V I G S I S G V R Y I L E T F P
16801 atacttttcccagGTAATTGGATCTATCTCTGGAGTTAGGTACATTCTTGAAACCTTTCC
116 E I G F G P R L N V D I H R G A L T E G
16861 TGAAATAGGATTTGGGCCGCGACTAAACGTGGACATCCATCGTGGTGCACTCACTGAAGG
136 V L T F A I V I I S L G L S R K I P G S
16921 AGTCCTTACATTCGCAATTGTTATCATCTCTCTCGGACTCTCAAGAAAGATCCCTGGTAG
156 F F M K T W I S S V S K L A L Q I L G S
16981 TTTCTTCATGAAGACTTGGATATCAAGCGTCTCCAAATTAGCTCTTCAGATACTGGGCTC
176 D L T G G C M N P A S
17041 TGATCTGACGGGCGGTTGTATGAACCCAGCTTCTgtaagtgttgtaaatatcattagtac
17101 gtgatgaataatacatcttagatatgttttccattgtcaaaaattttgaaacagagatga
187 V M G W A Y A R G D
17161 actagtatgtatgggtttttaaaatgtgtagGTGATGGGATGGGCTTACGCCCGTGGAGA
197 H I T K E H I I V Y W L A P I E A T V L
17221 TCATATAACCAAGGAGCATATAATTGTATATTGGCTTGCTCCAATAGAAGCAACTGTTCT
217 A V W T F K L L V R S R K Q E K K G K S

17281 GGCAGTGTGGACATTTAAGCTGCTTGTTCGTAGCCGAAAGCAAGAGAAGAAGGGTAAATC
237 D *
17341 AGAT**TGA**tgatttttgccctctaggtttgatgaagaatgatcaaacagaagtaattcact
17401 gtggtttctaattgtagagctctttcatttatcttctttttgctcaacagcctatcagat
17461 caactgtgttagcctctgctggagacttttaactaatgtaaatacttttattattttatg
17521 cataataactatcacttacgcatccattatttctggaaagtaacttaaatattttttttg
17581 taaaaaatattttatttggtcaatttcaatgtcaattcttagtgctttaaa
