# Supplementary material for: Genome-wide comparison reveals divergence of cassava and rubber aquaporin family genes after the recent whole-genome duplication
Source: BMC Genomics. 2019 May 15;20:380. doi: 10.1186/s12864-019-5780-4 (PMC6521647; doi:10.1186/s12864-019-5780-4)
Supplement: Supplementary file 3 — The gene model for MeNIP1;1. (PDF 78 kb) [file 12864_2019_5780_MOESM3_ESM.pdf]

Additional file 3: The gene model for *MeNIP1;1*. The coding region is marked with uppercase letters, under which are its deduced amino acids. The transcribed untranslated regions, including 5' UTR, intron and 3' UTR sequences, are marked with lowercase letters. The start and stop codons are marked with bold letters.

1 caaagcgaatgggctgaattccttcctggttctgagctgggccggaagataaggttaaga

61 ctagacccagaggggatctgttcattgggccgaaagagctgggccggcctgattgaagaa

121 tctgactgaagcccggtctttgagctgagcgggctggacctcgctcttggaggagactta

181 gaagccctcagattatgggctgtcctaatgggcctgatcttagacggggcgaaaaaatcc

241 agcggtcatcagattcgatttgaaaaagaaagacacgaaaccaatgtcaatcttcatccc

301 tttctcagttttccattttcacaatgaaatcaataacaatctcaattacaatacaatttc

361 acaattcaaaatgaaaaaaatactctcaaataaagaggaagaaatacaccatcaaagact

421 taaatgcatagatcaagaaactctcaaacacaagttatttatatagagagttatatagac

481 ctaaacataaacactatggagagcagcggtccatttccactgcagggagcagcagcaagc

541 agcaataatgaggtgggcttcttcacagaaacgacgagccggcgcttcttcgcagccgta

601 atgatggcaacgaggggggcggacttctttgcaagcggcaatgatgagagcttcttttacc

661 caagctccgcgacaacgaggaggccgcagagatgacaaggagaaaagggaggagaaggtc

721 aacttgaggaaagaaaaagagagaggatagggaggagggaggttgtacaaatggatggtg

781 ggtagattttgtaatgttctttatttatgaggctgtgggttcagtttttttattgatttc

841 agttagtcaaaaatcaaaatattaaaaaattaataatcgaatcaaattaaaaagattaaa

901 taactgaaccaatcaacttgacttgattggattggatttgcttttcagttaaaaaccaaa

961 aactgttgagccctaacgtgttgttttcagtctaaccttaactaataatagaaagaccag

1021 tttcaaaaataataatatgaaaatcccaagaaagacaaggacctgaggaggctgtatttg

1081 atcaatctctgccatttcatattctgaaaaagtctaattcatcgtctcttctctcctttg

1141 attttggatctgtcttcacttataaatactaatacgtgttatccctaatttgtcgcaaaa

1201 cactttggtgagaaagccatatgctgagcaatatatatctttgcttctcttatttgctcg

1261 cttcttactttctgattgttttgtgaattaaattcttctacttagacgaaaaaataaaaa

1 M A N D Q N K I V L D V K D D

1321 caaaaaaaagtagtc**ATG**GCTAATGACCAGAATAAAATTGTGTTGGATGTTAAGGATGAT

16 N H C T S L P P C K D I P R C A S D K G

1381 AATCATTGCACTTCTCTTCCACCTTGTAAGGACATTCCACGCTGTGCATCCGATAAGGGA

36 D S N L S I S V P F I Q K

1441 GATTCAAACTTAAGCATCTCTGTACCTTTCATTCAAAAGgtgaattactgatctttcaat

1501 ttaaatgttagttcttatcttggacatggttttctttcaaaaatcttggatagtttcaag

1561 gctatgttgctttatttgccattaaagattgaataatatctgatttttttatgttcatga

49 L I A E M I G T Y L L V F T G

1621 gtgatcgatgtgcagTTGATAGCTGAGATGATAGGCACGTATTTATTGGTATTCACCGGA

64 C T A G S V N L N F D K V V T L P G V S

1681 TGTACAGCGGGGTCGGTGAACCTGAACTTCGACAAGGTGGTGACACTTCCAGGAGTATCG

84 I V W G L A V M V L I Y S V G H I S G A

1741 ATAGTTTGGGGATTGGCTGTGATGGTCTTGATTTACTCTGTTGGCCATATCTCTGGTGCT

104 H F N P A V T L A F A T C K R F P C K E

1801 CATTTCAACCCTGCTGTCACTCTTGCCTTTGCCACCTGCAAGAGATTTCCCTGTAAAGAG

1861 gtaaaaccaaactaaaactataccttgttgaaaataacgtaaactacacatacccaggtg
1921 ggaaaccttgagtttcccgaaattgtttcaaaaaaaaaaaaacaaaaagagatcaataca
1981 atcaagctgttgatcaattgagcttagacagaaaatctttgaagtgttgatattcttttt
124 V P A Y I A S Q V V G
2041 aatccctcttttcttatctggtaatgtagGTGCCTGCTTATATAGCAAGTCAAGTCGTTG
135 A T L A A G T I R L I F P G K Q D Q F V
2101 GTGCAACACTGGCAGCTGGAACGATTAGATTAATTTTCCAGGGAAGCAAGATCAATTTG
155 G T M P T G S D M Q S F V I E F I I T F
2161 TAGGAACAATGCCTACTGGTTCAGACATGCAATCCTTTGTAATTGAGTTCATAATCACTT
175 Y L M F V I S G V A T D N R A
2221 TCTATCTCATGTTTGTCATATCAGGTGTCGCTACTGATAATCGAGCTgtaagttgttatt
2281 cttaaaacttgtttggctgaggttaagtagactaagaaagtagaacttccaacgcagtac
2341 tcagtatttgctgccattctttttgattgtcattagccaatagagccaatgaagctacc
2401 aacttcttgagctgtgatgatttcttagtatattacttggaattttgaattatttgactc
190 I G E L A G L A V G A T I L L N
2461 taacgtttatgcagATTGGTGAACTTGCTGGACTTGCTGTAGGTGCAACAATTCTCCTAA
206 V M I A G
2521 ATGTGATGATTGCTGGgtattagcctaaccgagtttacttataatactaaatctaattaa
2581 gaactaatcaaaattatccaaaatcattgactatgggtaacataaattaatttattttat
211 P I
2641 aagtgaaaatggataccctttcgatggatttgctgttatgactttgcaaaacagGCCAAT
213 S G A S M N P A R S L G P A I M S W Q Y
2701 TTCAGGAGCATCAATGAATCCAGCACGAAGCTTGGGGCCTGCAATTATGTCATGGCAATA
233 K G L W I Y I I S P I L G A Q A G A W S
2761 CAAGGGGCTATGGATTTATATTATATCGCCAATTCTTGGTGCACAAGCAGGTGCATGGTC
253 Y N I V R Y T D K P L R E I T K S A S F
2821 TTATAATATAGTTAGATATACAGATAAGCCTCTGAGAGAGATCACAAAGAGTGCCTCATT
273 I K S R A R H *
2881 CATCAAGAGCAGAGCACGTCAT**TGA**caagaacataatgtttttcatgtctaattgtgtt
2941 tcatgtcagagttaat
