# Supplementary material for: Genome-wide comparison reveals divergence of cassava and rubber aquaporin family genes after the recent whole-genome duplication
Source: BMC Genomics. 2019 May 15;20:380. doi: 10.1186/s12864-019-5780-4 (PMC6521647; doi:10.1186/s12864-019-5780-4)
Supplement: Supplementary file 4 — The gene model for MeXIP3;1. (PDF 96 kb) [file 12864_2019_5780_MOESM4_ESM.pdf]

Additional file 4: The gene model for *MeXIP3;1*. The coding region is marked with uppercase letters, under which are its deduced amino acids. The intron sequences are marked with lowercase letters. The start and stop codons are marked with bold letters.

```
   1 M  A  A  I  V  Q  D  E  E  S  L  S  V  N  K  I  Q  P  L  A
   1 ATGGCTGCAATTGTTCAAGATGAAGAAAGCCTCTCTGTGAATAAAATTCAACCCCTTGCC
  21 S  T  P  M
  61 TCTACTCCAATgcaagtgtcaaactttgttttctgtttaaccttttttttctattatttg
 121 gtttcagctcaaactctaactcgatgcatcacagttctgaaaataactctttgatttttt
  25                                                  A  E  Y
 181 ttttctttattaatctctataatcttattcatgtcatgaccaatgttacagGGCAGAGTA
  28  L  K  D  R  E  G  K  K  Q  C  N  A  T  R  L  K  K  V  L  G
 241 TTTAAAGGACAGGGAAGGGAAGAAGCAATGCAACGCCACTAGGCTCAAAAAAGTATTAGG
  48  V  E  D  F  F  S  L  M
 301 CGTTGAAGATTTTTTCTCTTTGATGgtaatgcacatatgcttttgtttagggtgtagttt
 361 catttatttaatacaaaattcagttgttttattcataaacaataaacaataaacatatta
 421 taattttgttaatagaaaaataatataagcataaaaatatctatgttaggcttttggaat
 481 gtctaaataccaaagggttatatgcatttttctttttattaatttatcaaaataattaag
 541 ttgatatctttatctactttttgtacattatatcaagtgcttatatggaaataaatatttt
 601 tgtacattgtatacatgaatcattttcattgaatttttaaaatatttttatttatttata
 661 atcattacattttaactgttacataaaaatttaaatttttattttatttattttttttatg
 721 gagtatttctcttaaattgatatagcaccattcagaaaaaatcttgagaatattttttgg
 781 catgctcattctgttagttaaattaagctaaattataatttctttgtagtttaacgaaac
 841 ctgtacattattttatgtatttttaaaagtcaactatttaattattgtaattttaaaaaa
 901 tcaataaactagtccctgcttgaaaaggatttttttttatttcactaaaatttagtatct
 961 atagttttatagatttcaacaatttaatatttatagtttaataaattctacacatttatt
1021 ttatattcttaaaaaattcagttcttgtaattttaaaacacaacaaagtacccttattat
1081 gaaattcaataagtgtaatttgatcttattatctgagcatgcatatatctagcgggctgt
1141 atttatcgatatttttttaataaaatgatataattttttttttctatttaatgatgaactg
1201 gaatataattatctccctttaataaattttatgcattaaaaaatctcgtagctaaaacat
1261 ttgagtttgagagttattaactaatacatcttttaatttttttaaaatatttttttaatat
1321 taaattcaaaaatttatagaatgaagcggatgaattatataaaatatttgcatagtacaa
1381 caaataaagttgagagagacttatataaaaatagttacactttttgaaaataaaaaaatt
1441 actaattaattatggtatttttaagttgtacggtttatttatttattacgacggttccgt
  56                                                 V  W  R
1501 ttataatttctcgcccaactttaccatacaaaattgtgttaactttttcagGTGTGGAGA
  59 A  S  M  S  E  F  L  G  T  A  V  L  V  F  A  I  D  T  I  V
1561 GCATCTATGTCAGAGTTCTTGGGCACAGCGGTTCTCGTTTTCGCAATAGACACCATTGTT
  79 I  S  T  I  E  S  E  T  K  L  P  N  L  I  L  S  I  L  V  A
1621 ATTTCCACAATTGAAAGTGAGACAAAATTACCAAACCTCATACTATCAATCCTAGTCGCC
  99 I  T  I  T  I  L  L  L  A  T  Y  P  I  S  G  G  H  I  N  P
1681 ATCACAATCACAATTCTCCTCCTGGCAACCTATCCCATTTCCGGAGGCCACATTAACCCC
 119 L  V  T  F  S  A  L  L  T  G  L  I  S  I  S  K  A  F  I  Y
```

1741 TTGGTCACCTTCTCAGCTCTACTCACCGGCCTCATTTCCATATCAAAAGCCTTCATATAC

139 I L A Q C A G G V V G A L A L K A V V N

1801 ATCTTGGCTCAATGTGCTGGTGGCGTTGTGGGTGCACTAGCACTAAAAGCTGTGGTCAAC

159 N N I E S T F S L G G C T L H I V A P G

1861 AACAACATTGAGAGCACATTTTCGCTTGGAGGCTGCACTCTGCATATTGTTGCACCAGGG

179 P N G P T V I G L E T G Q A L W L E I I

1921 CCAAATGGTCCTACTGTGATTGGGTTAGAAACTGGGCAGGCCCTTTGGCTAGAGATAATA

199 C G F V F L F A S V L M A F D H R Q A K

1981 TGTGGGTTCGTGTTTCTTTTTGCGTCAGTGCTGATGGCCTTTGATCATCGTCAAGCCAAG

219 A L G H V T I F T I V G I V L G L L V Y

2041 GCCTTGGGTCATGTCACAATTTTCACGATCGTGGGGATAGTGCTGGGTCTTCTTGTGTAT

239 V S T S V T T A K G Y A G A G L N P A R

2101 GTTTCGACTTCAGTGACAACAGCTAAAGGCTATGCTGGAGCTGGATTGAATCCAGCTAGG

259 C L G P A L V R G G H L W D G H W V F W

2161 TGTTTGGGTCCAGCATTAGTTCGAGGAGGTCATCTTTGGGATGGGCATTGGGTGTTTTGG

279 L G P A I S A V A F S L Y K K I I P P Q

2221 TTGGGACCTGCTATTTCTGCCGTTGCATTTTCTTTGTACAAAAAAATTATTCCACCTCAG

299 L S H T V L *

2281 CTTTCTCACACTGTTTTG**TAA**
