# Supplementary material for: Genome-wide comparison reveals divergence of cassava and rubber aquaporin family genes after the recent whole-genome duplication
Source: BMC Genomics. 2019 May 15;20:380. doi: 10.1186/s12864-019-5780-4 (PMC6521647; doi:10.1186/s12864-019-5780-4)
Supplement: Supplementary file 5 — The gene model for MeXIP3;2. (PDF 100 kb) [file 12864_2019_5780_MOESM5_ESM.pdf]

Additional file 5: The gene model for *MeXIP3;2.* The coding region is marked with uppercase letters, under which are its deduced amino acids. The intron sequences are marked with lowercase letters. The start and stop codons are marked with bold letters.

1 M A A I E E I V Q D E E I L S M N K M Q
1 **ATG**GCTGCAATTGAAGAAATTGTTCAAGATGAAGAAATCCTCTCTATGAATAAAATGCAA
21 P F P S T P M
61 CCCTTTCCCTCTACTCCAATgcaagtgccaaattatatatgtttcttttttttttttta
121 gctataattaattttgactagtatttgaacttgagaaattagttctgaagacaacttatt
181 actggattcaagttcattgattcctttaattttctttttctgttcttttttattacctta
28 G G H L Q D K E G K M Q C
241 tcatatcactaatgttacagGGGAGGGCATTTACAAGACAAGGAGGGAAAAATGCAATGC
41 N F T R L G K I L G L E E L F S L M
301 AACTTCACTAGACTGGGAAAAATATTAGGCCTTGAAGAATTGTTCTCTTTGATGgtaatc
361 acatgtgcatttgtttggagtgtagtttattatctcatctatataatttattttattaaa
421 aaaattgattgttctattcatgattagtagaaaataagaatattataatttttctcaata
481 gaaaattaaaaaattggaagctttattttatgaaatttaataatgtggaagggattaatg
541 acatttgagtaaacattaattgatacttgttatgcgtttggaatgcctgaataccaaagg
601 gttatatgcatttttcttttaattaacttattttatttttgcacataaagacctactcgc
661 catattagttgttctaatagtggttagttgttttaatagcaaacaattgtattactagca
721 attaacttttagttattaactattaatgcttagttgttaaatgtgaaattaaaaaaaaaa
781 aaagaagccaaagatgaaaaagtgataataaatgaaaatttatatattaaaggataagct
841 tataaatttataaaaaagacaaggatataagagttaaaaaaaataatccgttgtggccaa
901 acactcaggggaaatggatttttaataatcacttattcaaattttaacactaatacaagt
961 gagtagccaccgaataataaataataaaaataatattttaactttatttaaacacagtgt
1021 attgtcactaaacactataatcgaacacatcctaaattcattaacgaaaatagctattat
1081 tgtgaattatacatatcaaataatattttcacagccattcacagatttagttagatggta
1141 aatatatcttagtaaatctaagaaatttaatattctattcctccacttctcaatctacat
1201 tttaagaaaaaatatatatatatatctttaccaaaaaaaatccctttacaatgtgcaaaa
1261 gttaaataatccttttataatgttattatttatatatttataccttttagttataatcca
1321 tgaatcatataataaataatagatctattatatttaagaaataatcatctatcagttaag
1381 taatacttctttttcactctaattaagaaactgcatttccaattatactaagattatgaa
1441 tcacaaatttaagaatctccctttttaacttaaaatccaccggccatagtttttttaataa
1501 tcaaatcttcatgcttgaaattgaatccttaaagctttgagtcttcaatcttcaaattgg
1561 gtgttgctaatttcttcaaaatctttacaccatcaattttcttaattttgtgtcaaatgt
1621 tttatctaaacatttacttttttttttttacacctcaagattatttacatgctttgatat
1681 caatttgttataataatcactcacaaatattaaaaaagaattccaaattttaacgtgatt
1741 cgtccttttaacatagagtcatgtgtaaaaacattcatctttcactatcttcgctaaaag
1801 ttcaaaactatctacctatcaatttgattagacttaattaatttattacaataaattagt
1861 tataatcctcaataaacagtccaatatatttactagaactatattatgtatcaaaagttg
1921 tctttatcgacatagcaaaacctccctcatcattagacaagaattctttttacttgaatt
1981 ctattccctttctccggcttaagcatgaaactgcgctggataaaagccaaataatttctt
2041 cataatggtattattagcattacaccttttagtcctattatattaaaaatcttactcaaa

2101 ttacgaataagccaataaataaaagatttactctatctataaaataattctttatctatt

2161 aaatatttctcacatcctaattagaaaagagcctttctaatcatattagaattacgagtc

2221 ataaatctaataaatattttaatttttaagtaattgggtataaaaatcaactttgtacaa

2281 taactttttaatacctttcaattccttaacattttctctacagtttcagtatacaaaata

2341 gtgttaacttttagaaaaattactattttattcttaagattttaggaaattaatgagctc

2401 attcctataatttttagacacaatcaaaagatctcttggaaagtgtttttctagtttat

2461 aaactagtcaaactagcttaattataaactgatcaaaataacttagaagctctaaaaata

2521 aattgatatgtttgtttatagtttttttacgagtttctaaactcagcttattgactagct

2581 tataagctagttaaattggcttatacactctaaaaataattcgacatgtttgtttataat

2641 atttttacaagttcctaagttcaacttataagttgaaaaaataaattcgggtaagtaga

2701 ttctcattttggtgcttataagtactatattattttaaaataaaaatactttttgtggca

2761 aaagattaccattaaatacgtgaaattcaccacttagtccaacaaaaaatgcgatgttaa

2821 attattttgatacttttctataaatttcaatatacctttagttgggactgttttctcact

2881 aaaattttgctataaaaaacttaatttgttatataagttgttatatttctcacaattaat

2941 tttaaatttttataaattttaaataacttataaatatttaatgcttacaaattgattttc

3001 ataaggtaagttaaataacctttttactattttgaaatacaactatttatttattctgtta

3061 gtatttttgttagtgatagaattaaataatttataataatgaaacatgagtgaccttata

3121 ataatttatttaaaatgtaaaggattaaatttaaaaggacattttgtgacttttatcaaa

3181 tgtttaatgaaatttgtcaatgtataaaatagaaggattaataatagcattcaaagtaat

3241 aagagattgttaattgggtttaagaattatatggataaattaattaatttattaagaaaa

3301 atttattaattatttttttattttaaaaaataaatttgaaatatttttatttttttaaaaaa

3361 tttactaactagttattccattaattttaattattaaatataaaaaagattaaattattt

3421 ttaataaagagaaagtttttataaatatcaaaattatttaatatattttttttaaattata

3481 taaattaaatagtaaaatatttatttattaaaattaaccggtaaattaattaataaattt

3541 tttacaatattaaaaatattttaatgatttttaaaaatgaagtaacaattaacgtatttt

3601 tatataatattgagattttttttttgaaatggatataatattgagattatatattaattt

59 V W

3661 ttccatttattaaaaactaaggaatctaatgataatcatccctcaactttttcagGTATG

61 R A S L S E F L G T A V L V F V I D T V

3721 GAGAGCATCTTTGTCAGAGTTCTTGGGCACAGCGGTTCTCGTCTTCGTAATAGACACTGT

81 V I S T V E S E T K V P N L I L S I L V

3781 AGTTATTTCCACCGTTGAAAGTGAGACAAAAGTACCAAATCTTATACTATCAATCCTTGT

101 A I T V T I I L L A T Y P I S G G P I N

3841 TGCCATCACCGTCACCATTATCCTGCTAGCAACTTATCCCATTTCCGGCGGCCCCATTAA

121 P L V T F S A L L T G L I C I S K A F I

3901 CCCTTTGGTCACCTTCTCAGCTCTACTCACCGGCCTCATTTGCATATCAAAAGCCTTCAT

141 Y I L A Q C A G G V V G A L A L K A V V

3961 ATACATCTTGGCTCAGTGTGCTGGTGGCGTTGTGGGTGCACTAGCACTAAAAGCTGTAGT

161 N S N I E S T F S L G G C T L H I V E P

4021 CAACAGCAATATTGAGAGCACATTTTCGCTTGGAGGCTGCACTCTGCACATTGTTGAACC

181 G P N G P T V I G L G T G Q A L W L E I

4081 GGGGCCAAATGGTCCTACTGTGATCGGGTTAGGGACTGGGCAGGCCCTTTGGCTGGAGAT

201 I C G F V F L F A S V L M A F D H R Q A

4141 AATATGTGGGTTCGTGTTTCTGTTTGCGTCAGTGCTGATGGCCTTTGATCATCGTCAAGC

221 K A L G H V K I F T I V G I V L G L L V
4201 CAAGGCATTGGGTCATGTTAAAATTTTCACAATCGTGGGGATAGTGCTGGGTCTTCTTGT
241 Y V S T S V T T A K G Y A G A G L N P A
4261 GTATGTTTCGACTTCGGTGACAACAGCTAAAGGCTATGCTGGAGCTGGGCTGAACCCCGC
261 R C L G P A I V R G S H L W D G H W V F
4321 TAGGTGTTTGGGTCCAGCAATAGTTCGAGGAAGTCATCTTTGGGATGGGCATTGGGTGTT
281 W V G P A V S A V V F S L Y T K I I P P
4381 TTGGGTGGGTCCTGCTGTTTCTGCCGTTGTATTTTCTTTGTACACAAAAATTATTCCACC
301 Q L S H T V F *
4441 ACAGCTTTCTCACACCGTTTTC**TAA**
