# Supplementary material for: Genome-wide comparison reveals divergence of cassava and rubber aquaporin family genes after the recent whole-genome duplication
Source: BMC Genomics. 2019 May 15;20:380. doi: 10.1186/s12864-019-5780-4 (PMC6521647; doi:10.1186/s12864-019-5780-4)
Supplement: Supplementary file 6 — Alignment of cassava AQPs with structure determined Spinach PIP2;1. (PDF 237 kb) [file 12864_2019_5780_MOESM6_ESM.pdf]

Additional file 6: Alignment of cassava AQPs with structure determined Spinach PIP2;1. Multiple sequence alignment was performed using MUSCLE. Transmembrane helices (TM1–TM6) and the two short helices forming the two NPAs (HB and HE) (shaded), P_1–P_5 residues (shown in blue), NPA motifs (shown in red), and ar/R selectivity filter residues (shown in green) are indicated. The highly conserved cysteine residues in XIPs are shown in **bold**. The positions corresponding to S115 and S274 in SoPIP2;1（Genbank accession number 1Z98）are highlighted in bright green. The residues corresponding to S262 in GmNOD26 (Genbank accession number P08995) are shown in sky blue. The residues at the position corresponding to L197 from SoPIP2;1, determined to be the key residue involved in gating (occurring just before TM5) are underlined.

```
SoPIP2;1        ----------------MSKEVSEEA-----------QAH-QHGKDYVDPPPAPFFD----
MePIP1;1        -------------MEGKEEDVRLGANKFTERQPIGTSAQT--DKDYKEPPPAPVFE----
MePIP1;2        -------------MEGKEEDVRLGANKFTERQPIGTSAQT--DKDYKEPPPAPLFE----
MePIP1;3        -------------MEGKEEDVTLGANKYRETQPIGTAAQSQDDKDYTEPPPAPLFE----
MePIP1;4        -------------MEGKEEDVRLGANKYRETQPIGTAAQTQDDKDYTEPPPAPLFE----
MePIP2;1        ----------------MAKDVEVGG-----------QGSGFQAKDYSDPPPAPLID----
MePIP2;2        ----------------MAKDIEVGG-----------HGSDFQAKDYHDPPPAPLID----
MePIP2;3        ----------------MVKDVTE--------------QGSFSGKDYHDPPPTPLFD----
MePIP2;4        ----------------MAKGITE--------------PGSFSSKDYHDPPPAPLID----
MePIP2;5        ----------------MAKDVEVAE-----------NPGEFSGKDYHDPPPAPLID----
MePIP2;6        ----------------MAKDVEVAE-----------NPGEFSAKDYHDPPPAPLID----
MePIP2;7        ----------------MAKDVTEET-----------QP--THGKDYVDPPPAPLID----
MePIP2;8        ----------------MAKEVSEET-----------QP--AHGKDYVDPPPAPLID----
MePIP2;9        ----------------MAKEMGEEGP----------VEHQQHAKDYVDPPPAPLLD----
MePIP2;10       ----------------MAKEVTEEA-----------GEASQQERDYVEPPPAPLFD----
MeTIP1;1        ---------------------------------------------MPIRNIAVGH-----
MeTIP1;2        ---------------------------------------------MPIRNIAVGH-----
MeTIP1;3        ---------------------------------------------MPVTRIAVGN-----
MeTIP1;4        ---------------------------------------------MPITRIAVGN-----
MeTIP1;5        ---------------------------------------------MAISSIAIGS-----
MeTIP1;6        ---------------------------------------------MAITSIAIGS-----
MeTIP2;1        -----------------------------------------------MARIAFGR-----
MeTIP2;2        -----------------------------------------------MARIDFGS-----
MeTIP2;3        -----------------------------------------------MARIAFGS-----
MeTIP3;1        ---------------------------------------------MSTRRYAFGR-----
MeTIP3;2        ---------------------------------------------MSTRTYAFGR-----
MeTIP4;1        -----------------------------------------------MAKIALGS-----
MeTIP5;1        -----------------------------------------------MAPASLNAR----
MeNIP1;1        -----MANDQNKIVLDVKDDNHCTSLPP--------------CKDIPR---CAS------
MeNIP1;2        MAEISGTNGRHVVVLDVKEDNPCNPLPP--------------CNEPPP---CPSR-----
MeNIP2;1        ----MATIDPNLNTSASMEDNLVSVENP--------------KSQLVQ---SFR------
MeNIP3;1        ----MASPNSVTTTEVSPKLQLPTKYS---------------VAAEAKARHSQNW-----
MeNIP3;2        -----MAPVSPSPAGYSPKPTLATDLS---------------TIEEGNDGNSPPQ-----
MeNIP3;3        -----MAAVSPSPV-HSPKRTVSIDLS---------------KIEEK-MANTPSQ-----
MeNIP5;1        ----MPENETGTPTA-SAPATPGTPGGPLFPSLR-IDSLSYDRKSMPR-CKCLPVNAPTW
MeNIP6;1        ----MDNNNEEIPSAPSTPATPGTPGAPLFGGLRGERSGGAIRKSLLKSCKCFSVEEWTL
MeNIP7;1        -----MKHLLEDQLPPSPRISINASSNSGLSRDCPDMGSTEMSIDGHGHVFANSCVSG--
MeXIP1;1        --------MDLSPQQDDN----NHHSFSKSLENCEAINNVTGTK-SPKTSFLVFVG----
MeXIP2;1        -----MAETSRVFEDEENGYGGRKVQPFASTPQPD-LGKTEGKKQYPTT-FSSILG----
MeXIP3;1        --------MAAIVQDEES-LSVNKIQPLASTPMAEYLKDREGKKQCNATRLKKVLG----
MeXIP3;2        -----MAAIEEIVQDEEI-LSMNKMQPFPSTPMGGHLQDKEGKMQCNFTRLGKILG----
MeSIP1;1        ------------------------------------------------------------
MeSIP2;1        ------------------------------------------------------------
```

```
                                  TM1
SoPIP2;1      ----------LGELKLWSFWRAAIAEFIATLLFLYITVATVIGHSKETVV-------CGS
MePIP1;1      ----------PGELSSWSFYRAGIAEFIATFLFLYITVLTVMGVSKSPNK-------CAT
MePIP1;2      ----------PGELSSWSFYRAGIAEFIATFLFLYITVLTVMGVSKSNNK-------CAT
MePIP1;3      ----------PSELTSWSFYRAGIAEFIATFLFLYISVLTVMGVVKAPTK-------CST
MePIP1;4      ----------PSELTSWSFYRAGIAEFIATFLFLYISVLTVMGVVKAPTK-------CST
MePIP2;1      ----------AEEFTQWSFYRAIIAEFIATLLFLYITVLTVIGYKSQTDP-AKNADACGG
MePIP2;2      ----------AEELTQWSFYRAIIAEFIATLLFLYITVLTVIGYKSQTDP-TKNADACGG
MePIP2;3      ----------AAELTKWSFYRALIAEFIATLLFLYITILTVIGYKSQTDP-AKNADSCGG
MePIP2;4      ----------PAELTQWSFYRALIAEFIATLLFLYITVLTVIGYKSQTDPSLKNSDSCGG
MePIP2;5      ----------VEELGKWSFYRALIAEFIATLLFLYITVLTVIGYKSQTDP-AKNSDACGG
MePIP2;6      ----------IEELGQWSLYRALIAEFIATLLFLYITVLTVIGYKSQTDP-AKNSDACGG
MePIP2;7      ----------MAEIKLWSFYRALIAEFIATLLFLYITVATVIGYKKQTDP-------CGG
MePIP2;8      ----------TAELKLWSFYRALIAEFIATLLFLYVTVATVIGHKKQTDP-------CGG
MePIP2;9      ----------LKELKRWSFYRALIAEFMATLLFLYVSVATVIGYKSQADA-------CDG
MePIP2;10     ----------PQELGLWSFYRAVIAEFIATLLFLYVTIATVIGYKKQTDP-------CAG
MeTIP1;1      ----------PQEATHPDALKAALAEFISTLIFVFAGEGSGMAFSKLTDNA-----ANTP
MeTIP1;2      ----------PQEATHPDALKAALAEFISTLIFVFAGEGSGMAFSKLTKNA-----ANTP
MeTIP1;3      ----------PGEASEPDAIRAALAEFFSMIIFVFAGEGSGMAFSKLTKNG-----SATP
MeTIP1;4      ----------PGEASQPDALRAAMAEFFSMIIFVFAGEGSGMAFSKLTDNG-----STTP
MeTIP1;5      ----------PAEAGQPDALKAALAEFISMLIFVFAGEGSGMAFNKLTDNG-----STTP
MeTIP1;6      ----------PAEAGQPDALKAALAEFISMLIFVFAGEGSGMAFGKLTNNG-----STTP
MeTIP2;1      ----------FDDSFSLGSFKAYIAEFISTLLFVFAGVGSAIAYNKLTGNA-----ALDP
MeTIP2;2      ----------FGDSFSVGSIKAYLSEFIATLLFVFAGVGSAIAYSKLTADA-----ALDP
MeTIP2;3      ----------FGDSFSVGSIKAYLSEFIATLLFVFAGVGSAIAYSKLTTDA-----ALDP
MeTIP3;1      ----------PDEATHPDSIRAALAEFVSTLIFVFAGEGSVLALDKLYKET-----GPPA
MeTIP3;2      ----------AEEAIHPDSLRASLAEFVSTLIFVFAGEGSVLALDKLYTET-----GSPA
MeTIP4;1      ----------QREATQPDCIKALIVEFITTFFFVFAGVGAAMAADKLVGG--------SL
MeTIP5;1      ----------FKHSVTPDALRSYLAEFISTFFYVFAVIGSAMASRKLMPG-------ADP
MeNIP1;1      ------DKGDSNLSISVPFIQKLIAEMIGTYLLVFTGCTAGSVNLNFDKV----------
MeNIP1;2      ------TKEDSILSISVPFIQKLIAEVVGTYFLIFAGCTSVAVNLNFDKV----------
MeNIP2;1      ------------KNYPPAFLRKVVAEVMATYLLVFVTCGAAAISASDEQR----------
MeNIP3;1      ------LFTDDASPSI---FQKVIAELLGTYILVFVGCGSALTDKIQQ------------
MeNIP3;2      ------ASTAEVTVSSIINACNMMAELMGTYVIIFIGCGSILIDIKYE------------
MeNIP3;3      ------DSTAEVTVFP-INAHKIMAELMGTYVIIFIGCGSLMIEVKYG------------
MeNIP5;1      --GQPHTCFTDFPSPDVSLTRKLGAEFVGTFILIFAATAGPIVNQKYNGA----------
MeNIP6;1      EEGRLPPVSCSLPPPPASLARKVGAEFIGTLILIFAGTATAIVNQKTQGT----------
MeNIP7;1      ---------CFPKGMDLNPARMVLAEMMGTFVLMFCVCGIIGNTQITRG----------Q
MeXIP1;1      ----------AYEFFSPEMWRAVLVELVATACLLFTLTICILSCLESREP----------
MeXIP2;1      ----------LQDLSSLRVWRASLAEVLGTAALVFAMDTIVISSYETETK----------
MeXIP3;1      ----------VEDFFSLMVWRASMSEFLGTAVLVFAIDTIVISTIESETK----------
MeXIP3;2      ----------LEELFSLMVWRASLSEFLGTAVLVFVIDTVVISTVESETK----------
MeSIP1;1      ----------------MGAIKSAINDVLLTFMWVFCSSMFGLFTSLIATALG--VQHQFW
MeSIP2;1      -------------MDSAVTLRLIISDFVISFMWVWSGALIKIFLNRFLGLG-----HHEP
                                   :..    ::
```

```
                   TM2                        HB                  TM3
SoPIP2;1    VGLLGIAWAFGGMIFVLVYCTAGISGGHINPAVTFGLFLA--RKVSLLRALVYMIAQCLG
MePIP1;1    VGTQGIAWAFGGMIFALVYCTAGISGGHINPAVTFGLFLA--RKLSLTRAVFYIIMQCLG
MePIP1;2    VGIQGIAWAFGGMIFALVYCTAGISGGHINPAVTFGLFLA--RKLSLTRALFYIIMQCLG
MePIP1;3    VGIQGIAWAFGGMIFALVYCTAGISGGHINPAVTFGLFLA--RKLSLTRALYYMVMQCLG
MePIP1;4    VGIQGIAWAFGGMIFALVYCTAGISGGHINPAVTFGLFLA--RKLSLTRALYYMVMQCLG
MePIP2;1    VGILGIAWAFGGMIFILVYCTAGISGGHINPAVTFGLFLA--RKVSLVRAILYMAAQCLG
MePIP2;2    VGILGIAWAFGGMIFILVYCTAGISGGHINPAVTFGLFLA--RKVSLVRAVLYMVAQCLG
MePIP2;3    VGILGIAWAFGGMIFILVYCTAGISGGHINPAVTFGLFLA--RKVSLVRAVMYMVAQCLG
MePIP2;4    VGILGIAWAFGGMIFVLVYCTAGISGGHINPAVTFGLFLA--RKVSLVRAVMYMVAQCLG
MePIP2;5    VGILGIAWAFGGMIFILVYCTAGISGGHINPAVTFGLFLA--RKVSLIRALLYMVAQCLG
MePIP2;6    VGILGIAWAFGGMIFILVYCTAGISGGHINPAVTLGLFLG--RKVSLIRALLYMVAQCLG
MePIP2;7    VGLLGIAWAFGGMIFILVYCTAGISGGHINPAVTFGLFLA--RKVSLIRAVAYMVAQCLG
MePIP2;8    VGLLGIAWAFGGMIFILVYCTAGISGGHINPAVTFGLFLA--RKVSLIRAVAYMVAQCLG
MePIP2;9    VGFLGVAWAFGGMIFILVYCIAGISGGHINPAVTFGLLLA--RKLSLVRAVAYMVFQCLG
MePIP2;10   VGLLGIAWSFGGMIFILVYCTAGISGGHINPAVTLGLFLA--RKVSLVRAIAYMVAQCLG
MeTIP1;1    AGLVAASLAHAFALFVAVSVGANISGGHVNPAVTFGAFVG--GNITLLRGILYWIAQLLG
MeTIP1;2    SGLIAASIAHAFALFVAVSVGANISGGHVNPAVTFGAFVG--GNITLLRGILYWIAQLLG
MeTIP1;3    AGLIAASLAHAFALFVAVSVGANISGGHVNPAVTFGAFIG--GNITLLRGILYWIAQLLG
MeTIP1;4    AGLIAASLAHAFALFVAVSVGANISGGHVNPAVTFGAFIG--GNITLLRGILYWIAQLLG
MeTIP1;5    AGLVAASVAHAFALFVAVSVGANISGGHVNPAVTFGAFVG--GHITLIRSILYWIAQLLG
MeTIP1;6    AGLVAASLAHALALFVAVSVGANISGGHVNPAVTFGAFVG--GHITLIRSVLYWIAQLLG
MeTIP2;1    AGLVAIAVCHGFALFVAVAVGANISGGHVNPAVTFGLALG--GQITILTGIFYWIAQLLG
MeTIP2;2    PGLVAVAVAHAFALFVGVAIAANISGGHLNPAVTLGLAVG--GNITILTGIFYWIAQCLG
MeTIP2;3    AGLVAVAVAHAFALFVGVAIAANISGGHLNPAVTFGLAVG--GNITILTGIFYWIAQCLG
MeTIP3;1    SGLVMIALAHALALFSAVSASINVSGGHVNPAVTFGALVG--GRISVLRAFYYWVAQLLG
MeTIP3;2    SGLVMVALAHALALFSAVSASINISGGHVNPAVTFGALVG--GRISVLRAFYYWVAQLLG
MeTIP4;1    AGLLFIALAHALVVAVMISAG-HISGGHLNPAVTLGLLAG--GHITVFRSILYWIDQLIA
MeTIP5;1    SSLVAVAIANAFALSSVVYVAANISGGHVNPAVTFSLAVG--GHISLPTALFYWISQMLA
MeNIP1;1    VTLPGVSIVWGLAVMVLIYSVGHISGAHFNPAVTLAFATC--KRFPCKEVPAYIASQVVG
MeNIP1;2    VTLPGISIVWGLAVMVLVYSVGHISGAHFNPAVTLAFATC--RRFPWKQVPAYIACQVTG
MeNIP2;1    ISKLGASIAGGLIVTVMIYAVGHVSGAHMNPAVTAAFAAV--RHFPWKQVPFYVAAQLTG
MeNIP3;1    LTIVGIAIVWGFVLMALIYALGHISGAHFNPAVSIALAAS--RKFSWKNVPIYILAQVLG
MeNIP3;2    ISAVGIAVAWGFVVMVMIYALGHVSGGHFNPAITIAFAVS--CKFPWRQVPGYVASQLTG
MeNIP3;3    ISPVGVAVAWGLVVMVMIYALGHVSGGHFNPAITIAFAIQ--CKYSWRKVLGYVASQIAG
MeNIP5;1    ESLIGNAACAGLAVMIIILSTGHISGAHLNPSLTIAFASL--RHFPWMQVPAYIAAQVSA
MeNIP6;1    ETLIGLAASTGLAVMIVILSTGHISGAHLNPSVTIAFAAL--KHFPWKHVPVYIGAQVMA
MeNIP7;1    VGLLEYASTAGLTVIILVFSLGPISGAHVNPAVTIAFAAF--GHFSWSRVPLYVLAQIVG
MeXIP1;1    EPKLLVPFAVFIIAFFFLLATVPLSGGHMSPVFTFIAALK--GVITLVRAIFYILSQCVG
MeXIP2;1    TPNLIMSALIAITVTVILNATSPISGGHINPVITLSAAFT--GLISLSRAAIYILAQCLG
MeXIP3;1    LPNLILSILVAITITILLLATYPISGGHINPLVTFSALLT--GLISISKAFIYILAQCAG
MeXIP3;2    VPNLILSILVAITVTIILLATYPISGGPINPLVTFSALLT--GLICISKAFIYILAQCAG
MeSIP1;1    VSLFITTFIVFVFVFLFGLIANLLGGASFNPTGTAAFYAAGFGEDNLFSMALRFPAQAAG
MeSIP2;1    RDEAIKAAFSIINMFFFAFLGKITNGGAYNPLTIFSSAISGDFSQFLLTVGARIPAQVIG
                .                       .*.  .*                   *   .
```

```
                TM3                                                TM4
SoPIP2;1        AICGVGLVKAFM-KGPYNQFGGGANSVAL-----------GYNKGTALGAEIIGTFVLVY
MePIP1;1        AICGAGVVKGFEGNRVYESLGGGANVVVS-----------GYTKGDGLGAEIVGTFVLVY
MePIP1;2        AICGAGVVKGFEGKRVYQTLGGGANVVAH-----------GYTKGDGLGAEIVGTFILVY
MePIP1;3        AICGAGVVKGFEGRQRYTILGGGANSVNS-----------GYTKGDGLGAEIVGTFVLVY
MePIP1;4        AICGAGVVKGFEGRKQYTLLGGGANSVNP-----------GYTKGDGLGAEIVGTFVLVY
MePIP2;1        AICGCGLVKAFQ-KAYYTRYGGGANELAD-----------GYSKGTGLGAEIIGTFVLVY
MePIP2;2        AICGCGLVKAFQ-KAYYNRYGGGANELAD-----------GYSTGTGLGAEIIGTFVLVY
MePIP2;3        AIAGAGLVKGFQ-NSYYKRYGGGANSLAD-----------GYSTGTGLGAEIIGTFVLVY
MePIP2;4        AISGVGLVKAFQ-RSHYKRYGGGANTLAD-----------GYSTGVGLGAEIIGTFVLVY
MePIP2;5        AICGCGLVKAFQ-KAYYNRYGGGANELSS-----------GYSKGTGLGAEIIGTFVLVY
MePIP2;6        AICGCGLVKAFQ-KAYYNRYGGGANELSS-----------GYSKGTGLGAEIIGTFVLVY
MePIP2;7        AICGVGLVKAFM-KHSYNGLGGGANSVAP-----------GYSKGTALGAEIIGTFVLVY
MePIP2;8        AICGVGLVKAFM-KHSYNGLGGGANSVAP-----------GYSKGTALGAEIIGTFVLVY
MePIP2;9        AIAGVGLVKAVM-EDSYSSLGGGANSVSS-----------GYSKGTALGAEIIGTFVLVY
MePIP2;10       AICGVGIVKGIM-KDFYNAQGGGANTVAD-----------TYSKGTALGAEIIGTFVLVY
MeTIP1;1        STVACLLLKFST-----GGLTTSAFALSS-----------GVGVWNAFVLEIVMTFGLVY
MeTIP1;2        STVACLLLKLST-----GGMATAGFALSS-----------GVGVWNAFVLEIVMTFGLVY
MeTIP1;3        SVVACVLLKFAT-----GGMETSAFALSS-----------GLSPWNAVVFEIVMTFGLVY
MeTIP1;4        SVVACLLLKFAT-----GGLETSAFALSS-----------GVSSWNAVVFEIVMTFGLVY
MeTIP1;5        SVVACLLLKFAT-----GGLETSAFALSS-----------GVGGGNALVFEIVMTFGLVY
MeTIP1;6        SVVACLLLKFAT-----GGLETSAFALSS-----------GVGAWNAVVFEIVMTFGLVY
MeTIP2;1        SIVACFLLKAVT-----GGLAVPTHGVAA-----------GVGAVEGVVMEIVITFALVY
MeTIP2;2        STVACLLLQFVT-----NGKSVPTHGVAY-----------GMNAFEGVVMEIVITFALVY
MeTIP2;3        SIVACLLLHLVT-----NGKSVPTHGVAS-----------GMNAFEGVVMEIVITFGLVY
MeTIP3;1        AIVASLLLRLVT-----NGMRPVGFYVAS-----------GVGEVHGLILEMVMTFGLVY
MeTIP3;2        SIVASLLLRLVT-----NGMRPVGFYVAS-----------GVGVVHGLILEMVLTFGLVY
MeTIP4;1        STVACLLLSYLT-----GGLATPVITLAS-----------GVGYAQGVVWEIVLTFSLLF
MeTIP5;1        SVMACLLLRVAT-----VGQSLPTYMIAE-----------EMTGFGATIIEGVLTFGLVY
MeNIP1;1        ATLAAGTIRLIFP-----GKQDQFVGTMP-----------TGSDMQSFVIEFIITFYLMF
MeNIP1;2        STLAAGTIRLIFT-----GKQDHFTGTMP-----------AGSDMQSFVVEFIITFYLMF
MeNIP2;1        AISASFTLKVLLH-----PIK-HVGTTSP-----------AGSDLQALIMEIVVTFSMMF
MeNIP3;1        ATLASLTLKVLFHDQ--HNIEATMTQYKD-----------STSDLEAIIWEFIITFILML
MeNIP3;2        STLAILTLSVMFHGK--ADIKFTVTQYLG-----------QATDLEGFMWEFITSFILMI
MeNIP3;3        STLAILTLSVMLHGR--ADIKFTVTQYSG-----------QATDLEGFIWEFITSFILML
MeNIP5;1        SICASFALKGVFH-----PFM-SGGVTVP-----------SVGVGQAFALEFLITFNLLF
MeNIP6;1        SMSAAFALKGIFH-----PIM-GGGVTVP-----------SGGYGEAFALEFIISFNLMF
MeNIP7;1        SILATYVGKCIY------GIKPELMATRP-----------LRDCNSAFWVEFIATFIIMF
MeXIP1;1        SIMAYMVIKSVMNNATVEKYSLGGCMID----GNGG----GIAPGTALVLEFSCTFVVLF
MeXIP2;1        AILGALALKAVVNSTIEQTFSLGGCTLSIVAPGPHGPIVVGLGTAQAFWLEIICTFVFLF
MeXIP3;1        GVVGALALKAVVNNNIESTFSLGGCTLHIVAPGPNGPTVIGLETGQALWLEIICGFVFLF
MeXIP3;2        GVVGALALKAVVNSNIESTFSLGGCTLHIVEPGPNGPTVIGLGTGQALWLEIICGFVFLF
MeSIP1;1        AVGGALAILEVIPPQYKHMLGGPTLKVDL---------------HTGAIAEGVLTFLITF
MeSIP2;1        SISGVRYILETFP-----EIGFGPRLNVD--------------IHRGALTEGVLTFAIVI
                .  .                                          .   *    * .
```

```
                                                  TM5                    HE
SoPIP2;1    TVFSATDPKRSARDS---HVPILAPLPIGFAVFMVHLATIPI--TGTGINPARSFGAAVI
MePIP1;1    TVFSATDAKRNARDS---HVPILAPLPIGFAVFLVHLATIPI--TGTGINPARSLGAAII
MePIP1;2    TVFSATDAKRNARDS---HVPILAPLPIGFAVFLVHLATIPI--TGTGINPARSLGAAII
MePIP1;3    TVFSATDAKRNARDS---HVPILAPLPIGFAVFLVHLATIPI--TGTGINPARSLGAAII
MePIP1;4    TVFSATDAKRNARDS---HVPILAPLPIGFAVFLVHLATIPI--TGTGINPARSLGAAII
MePIP2;1    TVFSATDPKRNARDS---HVPVLAPLPIGFAVFMVHLATIPV--TGTGINPARSFGAAVI
MePIP2;2    TVFSATDPKRNARDS---HVPVLAPLPIGFAVFMVHLATIPV--TGTGINPARSLGAAVI
MePIP2;3    TVFSATDPKRNARDS---HVPVLAPLPIGFAVFMVHLATIPI--TGTGINPARSLGAAVI
MePIP2;4    TVFSATDPKRSARDS---HVPVLAPLPIGFAVFMVHLATIPI--TGTGINPARSLGAAVI
MePIP2;5    TVFAATDPKRNARDS---HVPVLAPLPIGFAVFMVHLATIPI--TGTGINPARSFGAAVI
MePIP2;6    TVFSATDPKRNARDS---HVPVLAPLPIGFAVFMVHLATIPI--TGTGINPARSFGAAVI
MePIP2;7    TVFSATDPKRSARDS---HVPVLAPLPIGFAVFMVHLATIPI--TGTGINPARSFGAAVI
MePIP2;8    TVFSATDPKRSARDS---HVPVLAPLPIGFAVFMVHLATIPI--TGTGINPARSFGAAVI
MePIP2;9    TVFSATDSKRKARDS---FVPVLAPLPIGFAVFVVHLATIPI--TGTGINPARSFGAAVI
MePIP2;10   TVFSATDPKRNARDS---HVPVLAPLPIGFAVFMVHLATIPI--TGTGINPARSFGAAVI
MeTIP1;1    TVYATAVDPKKG------NLGIIAPIAIGFIVGANILAGGAF--DGASMNPAVSFGPALV
MeTIP1;2    TVYATAIDPKKG------SLGIIAPLAIGFIVGANILAGGAF--DGASMNPAVSFGPALV
MeTIP1;3    TVYATALDPKKG------NVGIVAPLAIGFIVGANILVGGAF--DGASMNPAVSFGPAVV
MeTIP1;4    TVYATAVDPKKG------NVGIVAPIAIGFIVGANILAGGAF--DGASMNPAVSFGPAVV
MeTIP1;5    TVYATAVDPKKG------DIGIIAPIAIGFIVGANILAGGAF--DGASMNPAVSFGPAVV
MeTIP1;6    TVYATAVDPKNG------NIGIIAPIAIGFIVGANILAGGAF--DGASMNPAVSFGPAVV
MeTIP2;1    TVYATAADPKKG------SLGIIAPIAIGFIVGANILAAGPF--SGGSMNPARSFGPAVA
MeTIP2;2    TVYATAADPKKG------NLGIIAPIAIGFIVGANILAAGPF--SGGSMNPARSFGPAVV
MeTIP2;3    TVYATAADPKKG------NLGIIAPIAIGFVVGANILAAGPF--SGGSMNPARSFGPAVV
MeTIP3;1    TVYATAVDPKRG------SLGIIAPLAIGFIVGANILVGGPF--DGASMNPARAFGPALV
MeTIP3;2    TVYATAVDPKRG------SLGIIAPLAIGLIVGANILVGGPF--DGASMNPARAFGPALV
MeTIP4;1    TVYGTIVDPKKG------AIDGLGPLLTGLVVGANILAGGSF--SGAAMNPARAFGPALV
MeTIP5;1    TIYAAG-DPRRS------LLGATGPLAIGLMAGANVLAAGPF--SGGSMNPACAFGSAVI
MeNIP1;1    VISGVATDNRAIG--------ELAGLAVGATILLNVMIAGPI--SGASMNPARSLGPAIM
MeNIP1;2    VISGVATDNRAIG--------ELAGLAVGATVLLNVMFAGAI--SGASMNPARSLGPAIV
MeNIP2;1    VTSAVATDTKAIG--------ELAGIAVGSAVCITSILAGPV--SGGSMNPARTLGPAIA
MeNIP3;1    SICAVATDHRASK--------DLTGVAIGGTLLVNALLAGPI--TGASMNPARSLGPAIV
MeNIP3;2    TICGVATDSRAIN--------ELSGVAVGAAILFDMLIAGRI--TGASMNPARSLGPALV
MeNIP3;3    TICGVATDSRAIN--------ELSGVAVGAAMLFDIIIAGKI--TGASMNPARSIGAALV
MeNIP5;1    VVTAVATDTRAVG--------ELAGIAVGATVMLNILVAGPS--SGGSMNPVRTLGPAVA
MeNIP6;1    VVTAVATDTRAVG--------ELAGIAVGATVMLNILIAGQS--TGASMNPVRTLGPAIA
MeNIP7;1    LSASLTFQKSIMH---------LSGFVVGLAIGLAVLITGPL--SGGSLNPARSLGPAII
MeXIP1;1    VGVTVAFDKRRFKELGLIMVCVILAASMGLAIFVSITVTGRAGYAGVGLNPARCLGPALL
MeXIP2;1    SSIWVAFDKRQAKPLGRVMVCSIIGVVVGLLVFISTTVTATKGYAGVGMNPARCLGPALI
MeXIP3;1    ASVLMAFDHRQAKALGHVTIFTIVGIVLGLLVYVSTSVTTAKGYAGAGLNPARCLGPALV
MeXIP3;2    ASVLMAFDHRQAKALGHVKIFTIVGIVLGLLVYVSTSVTTAKGYAGAGLNPARCLGPAIV
MeSIP1;1    AVLVIILRGPRNS--------LVQHWLLAVVTVTLVVLGAKY--TGPSMNPANAFGWAYI
MeSIP2;1    ISLGLSRKIPGS--------FFMKTWISSVSKLALQILGSDL--TGGCMNPASVMGWAYA
                                     .                    *   :**.   :* *
```

```
                                   TM6
SoPIP2;1        FNSNKVWDDQWIFWVGPFIGAAVAAAYHQYVLRA-------AAIKALGSFRSNPTN----
MePIP1;1        FNKDHAWNDHWVFWVGPFIGAALAALYHQIVIRA-------IPFKARA------------
MePIP1;2        FNKDHAWDDHWIFWVGPFIGAALAAVYHQIVIRA-------IPFKARA------------
MePIP1;3        FNKDKGWDDHWIFWVGPFIGAALAALYHQVVIRA-------IPFKK--------------
MePIP1;4        FNNDKGWDDHWIFWVGPFIGAALAALYHQVVIRA-------MPFKK--------------
MePIP2;1        YNQDKAWDDQWIFWVGPFIGAAIAALYHQYILRA-------GAVKALGSFRSTSNI----
MePIP2;2        YNQDKAWDDQWIFWVGPFIGAAIAAFYHQYILRA-------SAAKALGSFRSSSNI----
MePIP2;3        YNQDKPWDDHWIFWVGPFIGAAIAAFYHQFILRA-------GAVKALGSFRSNPTV----
MePIP2;4        YNQDKAWDDQWIFWVGPFIGAAIAAFYHQFILRA-------GAVKALGSFRSNPTV----
MePIP2;5        YNKDKAWDDQWIFWVGPFIGAAIAAFYHQYILRA-------AAIKALGSFRSNA------
MePIP2;6        YNEDKAWDDHWIFWVGPFIGAAIAAFYHQYILRA-------AAIKALGSFRSNA------
MePIP2;7        YNNDKVWDDHWIFWVGPFIGALAAAAYHQYILRA-------AAIKALGSFRSNPTN----
MePIP2;8        YNNGKIWDDHWIFWVGPLIGALAAAAYHQYVLRA-------AAIKGLGSFRSNATN----
MePIP2;9        YNNKTVWDDHWIFWAGPFLGAMAAAGYHQYILRA-------GAAKALGSFRSNPSI----
MePIP2;10       YNNDKAWDDQWIFWVGPFVGALAAAIYHQHILRA-------TAIKALGSFTTT-TN----
MeTIP1;1        SWS---WENHWVYWAGPLVGGGLAGLIYEFIFIG------NNTHEQLPTTD--Y------
MeTIP1;2        SWS---WENHWVYWAGPLIGGGLAGLIYEFIFIG------HNTHEQLPTTD--Y------
MeTIP1;3        SWT---WTSHWVYWVGPLIGAAIAALVYDTVFVG------ENAHEPLSTND--F------
MeTIP1;4        SWT---WTNHWVYWVGPLVGAAVAAIVYDNIFIG------PNAHEPLSTND--F------
MeTIP1;5        SWT---WDNHWVYWLGPFLGAGIAAVVYEMFFIS------PTTHEQFPSGD--F------
MeTIP1;6        SWT---WDNHWVYWLGPFVGAGIAAVVYEVFFIS------PSTHEQLPSAE--F------
MeTIP2;1        SGN---FHDNWIYWVGPLIGGGLAGLIYGNLYI---------PSDHAPLSNE-F------
MeTIP2;2        SGD---FSQNWIYWVGPLIGGGLAGVVYGQIFIG--------SYVPAPSSED-YA-----
MeTIP2;3        SGD---FSQNWIYWLGPLIGGGLAGLVYGQIFIG--------PYSAAPSSDD-FA-----
MeTIP3;1        GWR---WRNHWIYWLGPFIGAGLAGLIYEYLVIPTEPLPHHTHHQPLAPED--Y------
MeTIP3;2        GWR---WRNHWIYWLGPFVGGGLAALIYEYMVIPTEPLPHHTHHQPLAPED--Y------
MeTIP4;1        SWN---WTHHWVYWVGPLIGGGLAGFIYENFFIT-------RSHLPLPNDEENY------
MeTIP5;1        AGR---FKNQAVYWVGPLIGGAVAGLLYDNVVFP---IQVPDSIRGISDNTG-L------
MeNIP1;1        SWQ---YKGLWIYIISPILGAQAGAWSYNIVRYTDKPLREIT-KSASFIKSRARH-----
MeNIP1;2        SRQ---YKGLWIYIVSPILGAQAGAWVYNMIRYTDKPLREIT-KSASFLKNTGRA-----
MeNIP2;1        SAH---YKGIWVYLIGPVVGTLLGAWSYNLIRVTDKPVQAISPRSFSFKLRRIRSKDMEA
MeNIP3;1        SGV---YKKLWVYIVAPIIGALAATLVYSVLRVPKPE--KPEENNKNIFNHLYVPAEP--
MeNIP3;2        SRT---FECLWVYVVAPILGMITASSAYSFIWLPP----ADKETTKSV------------
MeNIP3;3        AKQ---FECLWVYIVAPILGMITASTMYCFIWLPSSENIVDKDNAKTV------------
MeNIP5;1        AGN---YRDLWIYLVAPTLGAVAGAGTYTLVKLRDDEADP---PRQVRSFRR--------
MeNIP6;1        ANN---YKGIWIYLTAPILGALCGAGTYSAVKLPEEDGDAHEKPSESRSFRR--------
MeNIP7;1        SWN---FKDIWVYITAPVIGSLAGALMFHALRVQRRPCNSTDSSSNADLLGHSIAFRS--
MeXIP1;1        HGG-SLWHGHWVFWVGPCLACTVYYVYTLTLPRD--------RMGSVDED----------
MeXIP2;1        RGG-HLWNGHWVFWVGPVIASVAFAVYTKIIPSA--------EVHA--------------
MeXIP3;1        RGG-HLWDGHWVFWLGPAISAVAFSLYKKIIPPQ--------LSHTVL------------
MeXIP3;2        RGS-HLWDGHWVFWVGPAVSAVVFSLYTKIIPPQ--------LSHTVF------------
MeSIP1;1        NKWHDTWEQFYVYWICPFIGAILAAWMFRLVFPP-------PAPKQKKA-----------
MeSIP2;1        RGDHITKEHIIVYWLAPIEATVLAVWTFKLLVRS-------RKQEKKGKSD---------
                            ::   *  .         .
```

```
SoPIP2;1        ---------
MePIP1;1        ---------
MePIP1;2        ---------
MePIP1;3        ---------
MePIP1;4        ---------
MePIP2;1        ---------
MePIP2;2        ---------
MePIP2;3        ---------
MePIP2;4        ---------
MePIP2;5        ---------
MePIP2;6        ---------
MePIP2;7        ---------
MePIP2;8        ---------
MePIP2;9        ---------
MePIP2;10       ---------
MeTIP1;1        ---------
MeTIP1;2        ---------
MeTIP1;3        ---------
MeTIP1;4        ---------
MeTIP1;5        ---------
MeTIP1;6        ---------
MeTIP2;1        ---------
MeTIP2;2        ---------
MeTIP2;3        ---------
MeTIP3;1        ---------
MeTIP3;2        ---------
MeTIP4;1        ---------
MeTIP5;1        ---------
MeNIP1;1        ---------
MeNIP1;2        ---------
MeNIP2;1        HNNDPLDAL
MeNIP3;1        ---------
MeNIP3;2        ---------
MeNIP3;3        ---------
MeNIP5;1        ---------
MeNIP6;1        ---------
MeNIP7;1        ---------
MeXIP1;1        ---------
MeXIP2;1        ---------
MeXIP3;1        ---------
MeXIP3;2        ---------
MeSIP1;1        ---------
MeSIP2;1        ---------
```
