# Supplementary figures and images for: Genome-wide comparison reveals divergence of cassava and rubber aquaporin family genes after the recent whole-genome duplication
Source: BMC Genomics. 2019 May 15;20:380. doi: 10.1186/s12864-019-5780-4 (PMC6521647; doi:10.1186/s12864-019-5780-4)

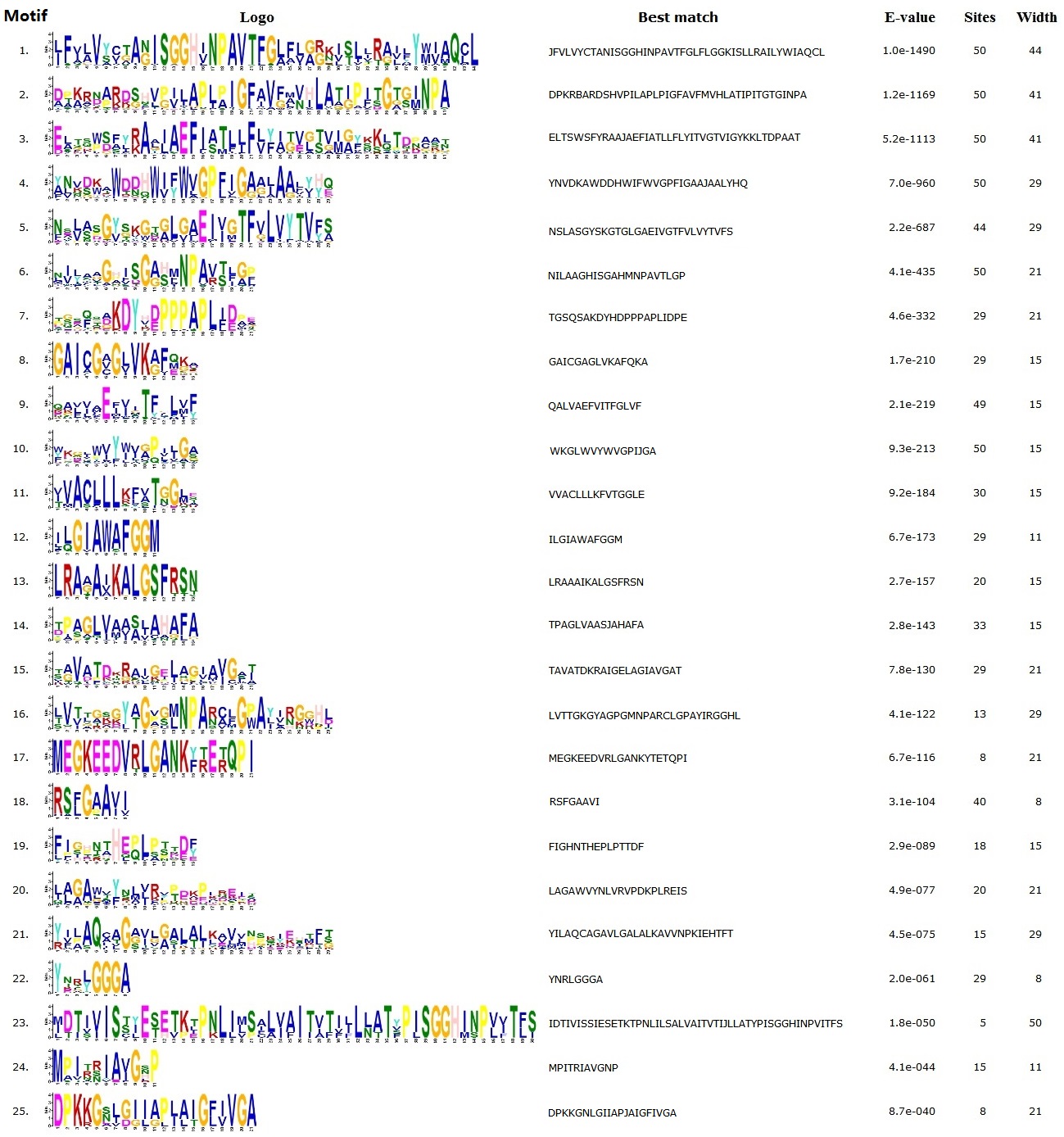

Supplement: Supplementary file 7 — Detailed information of 25 motifs identified in this study. (JPG 409 kb) [file 12864_2019_5780_MOESM7_ESM.jpg]

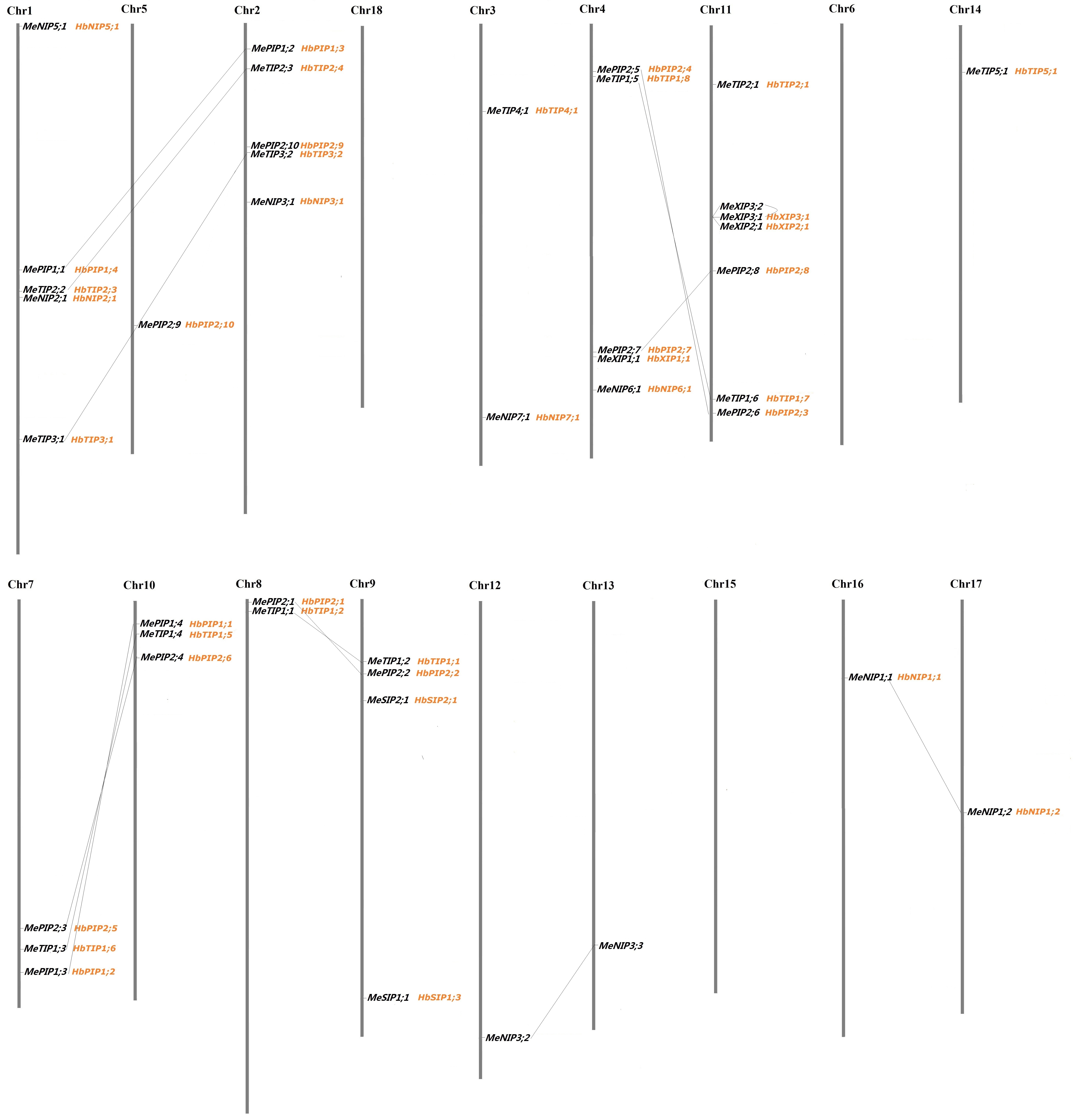

Supplement: Supplementary file 9 — Matched positions of 39 HbAQP genes on cassava chromosomes. The positions were based on synteny analysis, where HbAQP genes were marked in orange just following their syntenic genes in cassava. (JPG 1561 kb) [file 12864_2019_5780_MOESM9_ESM.jpg]
